# Daily co-trimoxazole prophylaxis to prevent mortality in children with complicated severe acute malnutrition: a multicentre, double-blind, randomised placebo-controlled trial

**DOI:** 10.1016/S2214-109X(16)30096-1

**Published:** 2016-06-02

**Authors:** James A Berkley, Moses Ngari, Johnstone Thitiri, Laura Mwalekwa, Molline Timbwa, Fauzat Hamid, Rehema Ali, Jimmy Shangala, Neema Mturi, Kelsey D J Jones, Hassan Alphan, Beatrice Mutai, Victor Bandika, Twahir Hemed, Ken Awuondo, Susan Morpeth, Samuel Kariuki, Gregory Fegan

**Affiliations:** a Kenya Medical Research Institute (KEMRI)/Wellcome Trust Research Programme, Kilifi, Kenya; b Centre for Tropical Medicine and Global Health, Nuffield Department of Clinical Medicine, University of Oxford, Oxford, UK; c University College, London, UK; d Imperial College, London, UK; e Mbagathi Hospital, Nairobi, Kenya; f Coast General Hospital, Mombasa, Kenya; g KEMRI Centre for Microbiology Research, Nairobi, Kenya

## Abstract

**Background:**

Children with complicated severe acute malnutrition (SAM) have a greatly increased risk of mortality from infections while in hospital and after discharge. In HIV-infected children, mortality and admission to hospital are prevented by daily co-trimoxazole prophylaxis, despite locally reported bacterial resistance to co-trimoxazole. We aimed to assess the efficacy of daily co-trimoxazole prophylaxis on survival in children without HIV being treated for complicated SAM.

**Methods:**

We did a multicentre, double-blind, randomised, placebo-controlled study in four hospitals in Kenya (two rural hospitals in Kilifi and Malindi, and two urban hospitals in Mombasa and Nairobi) with children aged 60 days to 59 months without HIV admitted to hospital and diagnosed with SAM. We randomly assigned eligible participants (1:1) to 6 months of either daily oral co-trimoxazole prophylaxis (given as water-dispersible tablets; 120 mg per day for age <6 months, 240 mg per day for age 6 months to 5 years) or matching placebo. Assignment was done with computer-generated randomisation in permuted blocks of 20, stratified by centre and age younger or older than 6 months. Treatment allocation was concealed in opaque, sealed envelopes and patients, their families, and all trial staff were masked to treatment assignment. Children were given recommended medical care and feeding, and followed up for 12 months. The primary endpoint was mortality, assessed each month for the first 6 months, then every 2 months for the second 6 months. Secondary endpoints were nutritional recovery, readmission to hospital, and illness episodes treated as an outpatient. Analysis was by intention to treat. This trial was registered at ClinicalTrials.gov, number NCT00934492.

**Findings:**

Between Nov 20, 2009, and March 14, 2013, we recruited and assigned 1778 eligible children to treatment (887 to co-trimoxazole prophylaxis and 891 to placebo). Median age was 11 months (IQR 7–16 months), 306 (17%) were younger than 6 months, 300 (17%) had oedematous malnutrition (kwashiorkor), and 1221 (69%) were stunted (length-for-age *Z* score <–2). During 1527 child-years of observation, 122 (14%) of 887 children in the co-trimoxazole group died, compared with 135 (15%) of 891 in the placebo group (unadjusted hazard ratio [HR] 0·90, 95% CI 0·71–1·16, p=0·429; 16·0 *vs* 17·7 events per 100 child-years observed (CYO); difference −1·7 events per 100 CYO, 95% CI −5·8 to 2·4]). In the first 6 months of the study (while participants received study medication), 63 suspected grade 3 or 4 associated adverse events were recorded among 57 (3%) children; 31 (2%) in the co-trimoxazole group and 32 (2%) in the placebo group (incidence rate ratio 0·98, 95% CI 0·58–1·65). The most common adverse events of these grades were urticarial rash (grade 3, equally common in both groups), neutropenia (grade 4, more common in the co-trimoxazole group), and anaemia (both grades equally common in both groups). One child in the placebo group had fatal toxic epidermal necrolysis with concurrent *Pseudomonas aeruginosa* bacteraemia.

**Interpretation:**

Daily co-trimoxazole prophylaxis did not reduce mortality in children with complicated SAM without HIV. Other strategies need to be tested in clinical trials to reduce deaths in this population.

**Funding:**

Wellcome Trust, UK

## Introduction

Severe acute malnutrition (SAM) contributes to 1 million childhood deaths annually worldwide and its treatment is a key target for reducing childhood mortality.^1^ Infectious disease is thought to be the main mediator of mortality in children with SAM. A short course of oral antibiotics given to children with uncomplicated SAM treated as outpatients reduced mortality and treatment failure in Malawi.^2^ However, a short course of antibiotics had no effect on these outcomes in Niger,^3^ although it did markedly reduce admission to hospital and improve early weight gain.

Children with SAM who have signs of infection or present with one or more Integrated Management of Childhood Illness danger signs,^4^ or do not pass an appetite test are classified as having complicated SAM, and WHO recommends that they should be initially treated in hospital—empirical antibiotics, treatment of specific medical conditions, and specialised therapeutic feeding are given.^5^ Once complications have resolved and appetite returns, children continue therapeutic feeding as outpatients.

**Research in context**

**Evidence before this study**

Children who are admitted to hospital with complicated severe acute malnutrition (SAM) are at greatly increased risk of mortality from common infections, including for a long period after discharge. Results from several randomised clinical trials in Africa have shown that daily co-trimoxazole reduces long-term all-cause mortality and readmissions to hospital among children who are susceptible to serious infections because of HIV. There is also evidence that antimicrobials improve growth. We aimed to investigate the efficacy of co-trimoxazole prophylaxis in children without HIV being treated for complicated SAM. We searched PubMed for randomised trials without date or language restrictions using the MeSH terms “child”, “malnutrition”, “mortality”, “clinical trials as topic”, “anti-infective agents”, or “anti-bacterial agents”. We identified two clinical trials of short-course antibiotic treatment in children with uncomplicated SAM treated as outpatients, but no previous clinical trials of longer-term antimicrobial prophylaxis to prevent mortality in children with complicated SAM after stabilisation. Generally, adequate evidence for the effectiveness of most interventions in children with SAM was absent or inconclusive.

**Added value of this study**

In our study, daily co-trimoxazole prophylaxis was well tolerated, but not effective at preventing mortality or morbidity, or improving nutritional recovery, in children aged 60 days to 59 months without HIV being treated for complicated SAM. However, some infections were prevented. Infants with complicated SAM had an especially high mortality during 1 year of follow-up, despite medical and nutritional care. Our results were different to those from trials of children with HIV, but similar to trials with children exposed to HIV but not infected. This outcome suggests that the interactions between antimicrobial prophylaxis, infections, and mortality depend on the specific immunopathology.

**Implications of all the available evidence**

SAM without HIV infection is not an indication for daily co-trimoxazole prophylaxis. To reduce mortality in children with complicated SAM will require a greater understanding of the condition and testing of other strategies in clinical trials.

Complicated SAM is typically associated with a high inpatient mortality.^6^, ^7^ However, even after provision of recommended treatment, apparent stabilisation, and discharge from hospital, children with SAM still retain a markedly increased risk of death. In Malawi, despite the provision of recommended medical and nutritional therapy, 17% of children with SAM (and without HIV) died within 1 year.^8^ In Bangladesh, among children with SAM and pneumonia, 9% died in hospital and a further 9% died within 3 months after discharge from hospital.^9^ Increased mortality after hospital discharge also occurs outside the context of SAM and is a generally recognised occurrence that might exceed inpatient mortality in resource-poor settings.^10^ In rural Kenya, children admitted to hospital had 7·7 times greater mortality in the year after discharge than community peers who were not admitted.^11^

Among children who are susceptible to infection because of HIV, daily co-trimoxazole prophylaxis reduced all-cause mortality and hospital admissions in two studies,^12^, ^13^ despite high levels of antimicrobial resistance being identified in vitro among invasive isolates at the study sites. Co-trimoxazole protected children with HIV against malaria, pneumonia, and sepsis.^14^ In other contexts, co-trimoxazole prevented recurrent urinary tract infections,^15^ pneumonia in children with measles,^16^ and infections in children with specific immune deficiencies.^17^ Co-trimoxazole is inexpensive, widely available, and has a known safety profile.^14^

Findings from a systematic review have shown beneficial effects of antimicrobials in children, including co-trimoxazole, on linear and ponderal growth, possibly by reducing nutrient diversion from subclinical infections and inflammation, and preventing or treating overt infections.^18^ Antibiotics are also widely used in animal husbandry to promote growth. However, important potential harms might occur from the long-term use of antibiotics, including toxicity and exacerbation of antimicrobial resistance. Hence, for their use to be justified, major potential benefits such as a reduction in mortality in high-risk groups of children should be targeted.^19^

We therefore tested our hypothesis that daily co-trimoxazole prophylaxis would reduce mortality and morbidity, and improve nutritional recovery, in children without HIV being treated for complicated SAM.

## Methods

### Study design and participants

We did a two-arm, multicentre, double-blind, randomised, placebo-controlled trial in four hospitals in Kenya (two rural hospitals in Kilifi, one urban hospital in Mombasa, and one in Nairobi). The study protocol is available in the supplement to this Article. All the study hospitals provided inpatient care for SAM, and outpatient therapeutic and supplementary feeding clinics supported by UNICEF or Concern Worldwide. In Kenya, *Haemophilus influenzae* type b conjugate vaccine was introduced in 2001 and pneumococcal conjugate vaccine (PCV, 10-valent) was introduced during this trial in March, 2011.

Children were eligible for inclusion if they were aged between 60 days and 59 months old, and had a diagnosis of SAM on the basis of mid-upper-arm circumference (MUAC) measurements (<11·5 cm for children aged ≥6 months and <11·0 cm for infants aged 2–5 months) or presence of kwashiorkor (oedematous malnutrition); had a negative HIV rapid-antibody test; and had completed the stabilisation phase of treatment as defined in WHO guidelines (appendix). All children admitted to the study hospitals were screened for eligibility by MUAC and examination for kwashiorkor. MUAC was chosen as the primary anthropometric criterion because of its high predictive value for mortality.^4^ Exclusion criteria were a known allergy to co-trimoxazole; if co-trimoxazole was specifically contraindicated; serious comorbidity likely to be associated with mortality unrelated to infection such as severe heart disease or malignancy; and residence outside the attending hospital's catchment area.

Children were enrolled after their parent or guardian provided written, informed consent and after medical and nutritional stabilisation—ie, in the rehabilitation phase of SAM treatment defined by WHO guidelines^5^ (additional details in the appendix). During the trial, enrolment was suspended during three long-term health-worker strikes and during several periods of political unrest. The Oxford Tropical Research Ethics Committee (Oxford University, Oxford, UK), the Ethical Review Committee (Kenya Medical Research Institute, Nairobi, Kenya), and the Expert Committee on Clinical Trials at the Pharmacy and Poisons Board (Nairobi, Kenya) reviewed and approved the protocol. Eligibility criteria, consent, serious adverse events, and study endpoints were verified against source documents for all participants by visiting monitors.

### Randomisation and masking

We randomly assigned eligible participants (1:1) to 6 months of either daily oral co-trimoxazole prophylaxis (given as paediatric water-dispersible tablets; 120 mg per day for age <6 months, 240 mg per day for age 6 months to 5 years) or matching placebo. The assignment was undertaken by the trial statistician (GF) with computer-generated randomisation of study numbers in permuted blocks of 20, stratified according to clinical centre and age older or younger than 6 months. Allocation was concealed in opaque sealed envelopes that were externally labelled with study numbers and used sequentially at each site. Treatment packs were likewise prelabelled with the study numbers according to the randomisation schedule. All patients, their families, and trial staff were masked to the treatment assignment. When twins or siblings were both enrolled, the first child was enrolled normally, then the second child was allocated by a trial statistician (GF) to the same arm by providing investigators with the next matching study number. Active and placebo blister-packed water-dispersible tablets were identical in appearance and dissolution.

The dose regimen of co-trimoxazole was that recommended by WHO for HIV care.^20^ This dose was used for children aged more than 6 months in the CHAP and ARROW trials^12^, ^13^ among children with HIV, in which efficacy against mortality and admission to hospital was shown (details of the resultant dosing in mg/kg are in the appendix). Active and placebo investigational products were tested for dissolution, and drug content at the National Quality Control Laboratory (Nairobi, Kenya) before the study began, and again during the last year of enrolment.

### Procedures

Baseline data on health, anthropometry, and socioeconomic status of probable prognostic importance were obtained by the study teams at each site. Children received standard care for SAM and other medical conditions according to WHO guidelines (2005) for complicated SAM.^5^ This care included the following empirical intravenous antibiotics: gentamicin (7·5 mg/kg intramuscular or intravenously) once per day for 7 days plus either benzyl penicillin (50 000 U/kg intramuscular or intravenously every 6 h) or ampicillin (50 mg/kg intramuscular or intravenously once per 6 h) for 2–7 days, changed to oral amoxicillin (25–40 mg/kg once per 8 h for up to 5 days) once medically stable. Therapeutic feeding was with lower protein and energy F75 milk during stabilisation and higher protein and energy F100 milk during the transition and rehabilitation phases,^5^ then ready-to-use therapeutic foods for children aged 6 months or more; and expressed breastmilk or dilute F100 for infants aged less than 6 months; advice on breastfeeding and complementary feeding was given.

While the children were in hospital, their parents or guardians administered the trial drug, supervised by the study team. The decision to discharge children from hospital was made by the clinical team and was not influenced by study procedures. At discharge, the study team ensured enrolment in a community-based, therapeutic-feeding programme, either at the hospital site or nearer to their home. A study fieldworker accompanied the family to their home at discharge to record an accurate route description, plot the household location using a handheld global positioning system, complete a home questionnaire, and obtain mobile-telephone numbers for the family members.

Scheduled follow-up after enrolment was once per month up to 6 months, then once every 2 months from 6 to 12 months (without study medication). We reimbursed fares to attend follow-up visits. Defaulters were traced by mobile phone or by home visit by fieldworkers. At each follow-up visit, anthropometric tests were done and remaining study drugs and empty blister packs were counted to assess adherence. A full blood count was done at enrolment, 2, 6, and 12 months. Besides regular follow-up, the study provided a telephone hotline, a free walk-in clinic, and met costs of readmission to hospital.

In the event of readmission to a study hospital, a standard admission clerking was undertaken and we did a blood culture, urine multiparameter dipstick and malaria rapid diagnostic test, or made up a blood slide. Urine was cultured when a clean sample was obtained and the dipstick indicated nitrites or leucocytes. For outpatient visits, tests were done as clinically indicated (laboratory methods are described in the appendix).

### Outcomes

The primary outcome was mortality during the 365 days of the study period. Deaths in hospital were documented by study clinicians, including the final diagnoses. For deaths outside the study hospital, we obtained a copy of the death or burial certificate from the family or village chief, examined copies of hospital or clinic records and, after a culturally appropriate period, a verbal autopsy was done. An endpoint review committee not directly involved in trial participant care (KDJJ and NM) reviewed all available information including medical and laboratory records and verbal autopsies for all deaths to assign causes while masked to treatment allocation.

Secondary outcomes were the frequency of non-fatal illness episodes resulting in readmission to hospital or outpatient attendance; the clinical syndromes associated with death or illness; pathogens detected from blood culture, urine culture, and malaria testing; suspected toxic effects during the period that investigational products were received; and changes in MUAC, weight-for-height, weight-for-length, weight-for-age, height-for-age, length-for-age, head circumference-for-age, and haematological indices.

### Statistical analysis

On the basis of data from a previous study of post-discharge mortality at one of the trial sites,^11^ we initially calculated that 800 children in each arm were needed to give power of more than 90% to detect a 33% reduction in mortality at 12 months from a baseline rate of 18 deaths per 100 child-years, at a two-sided α-level of 0·05 assuming 15% loss to follow-up. In April, 2011, the protocol was amended with respect to MUAC eligibility criteria to align with revised national protocols based on WHO guidelines and to simplify enrolment of infants younger than 6 months. We thus recalculated the necessary sample size to be 925 per arm on the basis of an expected reduction in the placebo arm event rate to 15 deaths per 100 child-years, because of the more inclusive MUAC criteria. In September, 2013, after an independent statistical review by Sarah Walker (Medical Research Council Clinical Trials Unit, London, UK) of the rate of events in the placebo group and actual loss to follow-up (5%), a decision was made to discontinue enrolment when at least 875 children per arm had been included, maintaining the original power and effect size.

A statistical analysis plan was reviewed and agreed by an independent Data and Safety Monitoring Committee (DSMC) and independent Trial Steering Committee (TSC) before unmasking the trial database. The DSMC oversaw the trial and reported to the TSC.

We did the analyses on an intention-to-treat basis and all statistical tests were two-sided. For the primary endpoint, we compared the two treatment allocation groups with respect to the primary endpoint using Kaplan-Meier plots and unadjusted proportional hazards models. We did prespecified subgroup analyses to assess heterogeneity of effect by age, kwashiorkor, and study period: the first 6 months (receiving study medication) and the second 6 months (off study medication). We assessed evidence of heterogeneity in the effect of randomised allocation across subgroups by comparing models including interaction terms, with models with main effects only, using log-likelihood ratio tests.

Morbidity events between randomised groups were compared by their incidence rate ratio (events per 100 child-years observed). We compared the distributions of continuous variables using student's *t* test, or Wilcoxon's rank-sum test if the variables were skewed. We compared categorical variables between randomised groups using χ^2^ tests. Trends in proportions across ordered groups were assessed by a non-parametric test for trend.

We did two post-hoc exploratory analyses to interpret the main findings. The first investigated effect modification by participants' receipt of conjugate pneumococcal vaccine, which was introduced during the trial. The second investigated effect modification by the presence or absence of the most common clinical syndromes—severe pneumonia and diarrhoea—at initial presentation. We did both analyses with log-likelihood ratio tests, as described earlier. This trial was registered at ClinicalTrials.gov, number NCT00934492.

### Role of the funding source

The funder of the study had no role in study design, data collection, data analysis, data interpretation, or writing of the report. The corresponding author had full access to all the data in the study and had final responsibility for the decision to submit for publication.

## Results

**Figure 1:**
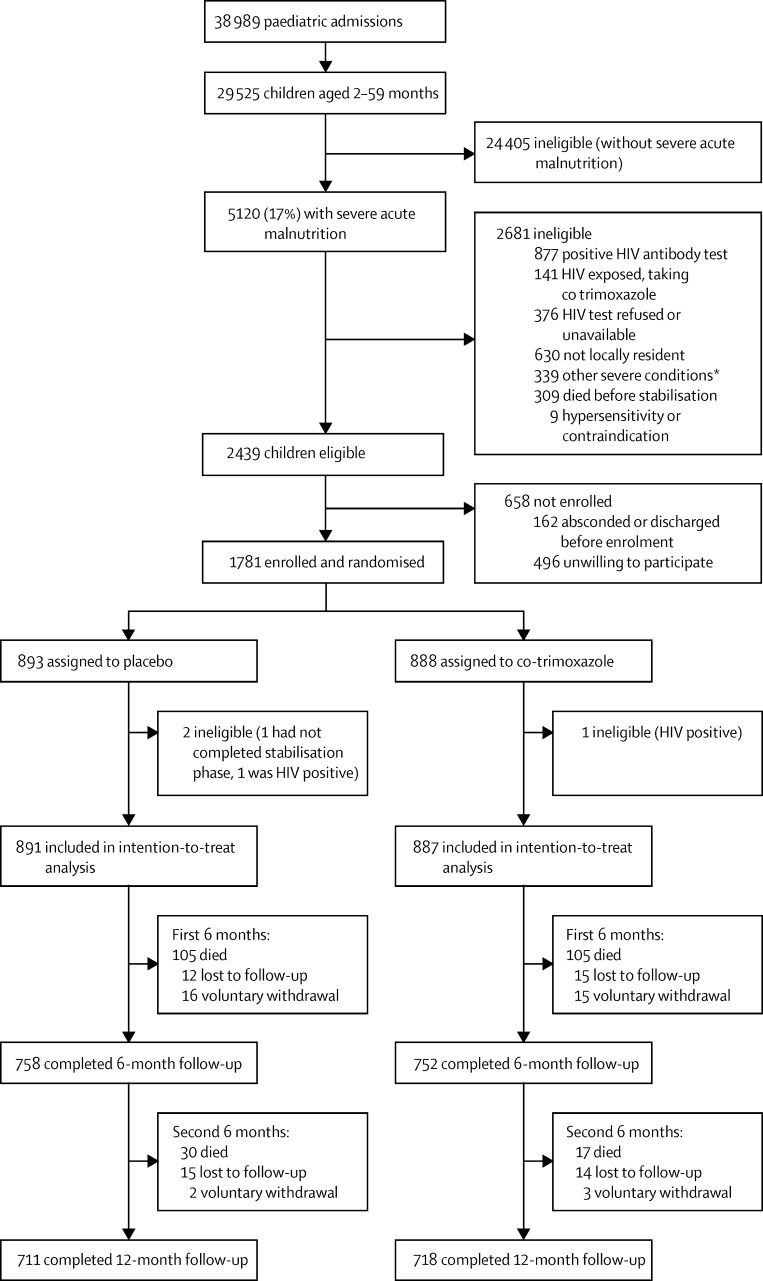
Trial profile *Includes severe congenital or acquired heart disease, malignancy, or probably terminal illness.

Between Nov 20, 2009, and March 14, 2013, from 2439 eligible children we recruited 1781 (figure 1, appendix). Three participants were withdrawn by the study team as ineligible soon after enrolment, without knowledge of their allocation. Thus, we analysed data from 1778 children: 887 assigned to co-trimoxazole prophylaxis and 891 to placebo.

The 1778 children had a median age of 11 months (IQR 7–16), 306 (17%) were younger than 6 months, 300 (17%) had kwashiorkor (appendix). Of the 1478 children without kwashiorkor, 1281 (87%) and 908 (61%) had weight-for-height *Z* scores of less than −2 and less than −3, respectively. Of 1778 children, 1221 (69%) and 784 (44%) had length-for-age *Z* scores of less than −2 and −3, respectively, and 230 children (13%) had clinical signs of rickets. At baseline, 1092 (61%) participants were breastfed, including 256 (84%) of the 306 infants younger than 6 months. Baseline characteristics were similar between the two groups (table 1).

**Table 1:** Baseline characteristics of the intention-to-treat population

		**Placebo N=891**	**Co-trimoxazole N=887**
Age (months)		10·8 (6·9–16·7)	11·2 (7·2–16·7)
Younger than 6 months		158 (18%)	148 (17%)
Sex			
	Female	428 (48%)	447 (50%)
	Male	463 (52%)	440 (50%)
Mother is primary caretaker		838 (94%)	823 (93%)
Caretaker completed primary education (n/total N)*		359/573 (63%)	347/577 (60%)
Currently breastfeeding		551 (62%)	541 (61%)
Received pneumococal conjugate vaccine†		524 (59%)	504 (57%)
Urban site		680 (76%)	676 (76%)
Nutritional oedema		149 (17%)	151 (17%)
MUAC (cm)		10·6 (1·09)	10·6 (1·05)
MUAC-for-age *Z* score ‡		−3·83 (1·05)	−3·81 (0·99)
Weight-for-length *Z* score§		−3·35 (1·26)	−3·32 (1·27)
Weight-for-age *Z* score§		−4·01 (1·12)	−3·96 (1·09)
Length-for-age *Z* score		−2·91 (1·67)	−2·82 (1·64)
Head circumference-for-age *Z* score*		−1·82 (1·47)	−1·73 (1·40)
Haemoglobin (g/L)		98·0 (21·4)	98·0 (22·4)
Clinical signs of rickets		129 (14%)	118 (13%)
Ocular signs of vitamin A deficiency		3 (<1%)	1 (<1%)
Cerebral palsy		34 (4%)	32 (4%)
Known tuberculosis at enrolment		31 (3%)	36 (4%)
Index admission for pneumonia		484 (54%)	474 (53%)
Index admission for diarrhoea		506 (57%)	515 (58%)
Treated for shock before enrolment		84 (9%)	100 (11%)
Impaired consciousness before enrolment		57 (6%)	56 (6%)
Days from admission to enrolment		6 (4–8)	6 (4–8)

Data are median (IQR), n (%), or mean (SD). MUAC=mid-upper-arm circumference.

* Data obtained from April, 2011.

† Received at least one dose of pneumococcal conjugate vaccine at enrolment.

‡ Excludes infants younger than 3 months, because no WHO (2006) reference exists for younger than this age.

§ Excludes children with kwashiorkor (oedematous malnutrition).

56 (3%) of 1778 children were lost to follow-up after a median of 186 days (IQR 66–303) and 36 (2%) voluntarily withdrew from further follow-up after a median of 73 days (IQR 47–126). We noted no differences between the groups in losses to follow-up or withdrawal (p=0·833). The total follow-up time was 763·3 child-years of observation (CYO) in the co-trimoxazole group and 763·3 CYO in the placebo group.

Adherence, measured on the basis of empty blister-pack counts, was a median of 95·5% (IQR 82·3–98·4%) in the co-trimoxazole group and 95·4% (74·2–98·8%) in the placebo group (p=0·666; appendix). Three children had a positive HIV-antibody test when readmitted to hospital, despite a negative test at enrolment (two assigned to placebo and one to co-trimoxazole). They were given open-label co-trimoxazole, maintained in follow-up, and included in the analysis on the intention-to-treat principle.

**Figure 2:**
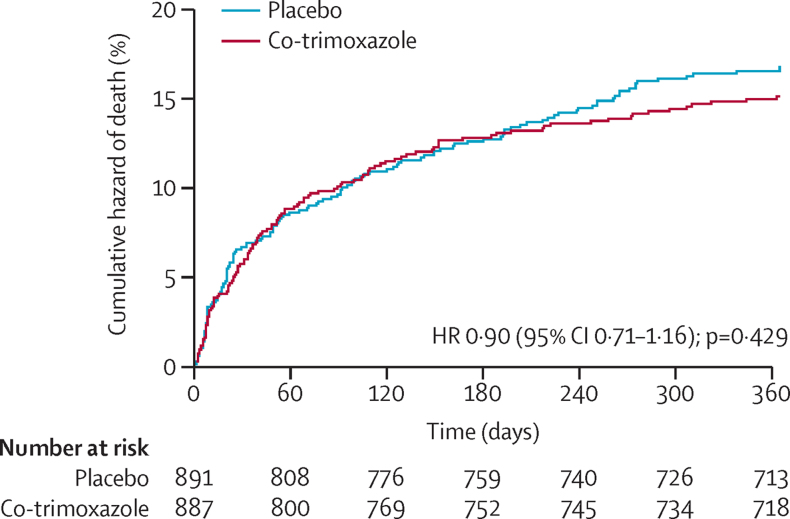
Cumulative hazard curves for time to death Kaplan-Meier curves are shown for time to death until 365 days. The time at risk observed was 763·3 and 763·3 child-years in the co-trimoxazole group and placebo group, respectively, of which 395·3 and 398·7 child-years, respectively, were during the first 6 months while receiving the study drug. HR=hazard ratio.

During 1527 CYO, 257 deaths occurred (14%, 16·8 per 100 CYO; 95% CI 14·9–19·0), 60 (23%) of which occurred during the index admission, 64 (25%) during a readmission to a study hospital, 29 (11%) in other hospitals, and 104 (40%) in the community (characteristics of children who died at different times are in the appendix). Mortality did not differ between the groups: 122 (14%) of 887 children in the co-trimoxazole group died, compared with 135 (15%) of 891 children in the placebo group (unadjusted hazard ratio [HR] 0·90, 95% CI 0·71–1·16, p=0·429; 16·0 *vs* 17·7 events per 100 CYO, difference −1·7 events per 100 CYO, 95% CI −5·8 to 2·4; figure 2). Results were similar for an analysis adjusted for age and site (adjusted HR 0·92, 95% CI 0·72–1·17; p=0·482).

No evidence was apparent for differences in mortality between intervention groups within prespecified fatal clinical syndromes, apart from the category of other infections comprising febrile illnesses other than severe pneumonia, diarrhoea, or sepsis (HR 0·58, 95% CI 0·36–0·92; table 2, appendix).

**Table 2:** Mortality (primary outcome)

		**Placebo (n=891)**	**Co-trimoxazole (n=887)**	**Hazard ratio (95% CI)**
All-cause mortality		135 (15%)	122 (14%)	0·90 (0·71–1·16)
Death from a specific syndrome*				
	Severe pneumonia	46 (34%)	49 (40%)	1·07 (0·70–1·60)
	Diarrhoea	25 (19%)	27 (22%)	1·08 (0·63–1·87)
	Clinically defined sepsis	27 (20%)	30 (25%)	1·11 (0·66–1·87)
	Other infections†	47 (35%)	27 (22%)	0·58 (0·36–0·92)‡
	Non-infectious causes§	5 (4%)	5 (4%)	0·98 (0·29–3·44)
	Unknown cause¶	22 (16%)	21 (17%)	0·96 (0·53–1·74)

Data are n (%) for all-cause mortality and n (% of deaths) for specific syndromes.

* The total for individual causes is greater than the number of deaths because more than one cause of death was identified in some children.

† Details are presented in the appendix.

‡ p=0·02.

§ Comprises three severe anaemia, two aspiration, one heart disease, one hepatic failure, one poisoning in the community, one drowning, and one during status epilepticus in a child with known epilepsy.

¶ Deaths in the community without sufficient information to determine cause.

There was no evidence for heterogeneity of effect by age group, the presence of kwashiorkor, site, or time period (appendix). In the 6 months after stopping study medication there was a non-significant reduction in mortality in the group assigned to co-trimoxazole (HR 0·56, 95% CI 0·31–1·02) and weak evidence of heterogeneity of effect between the two periods (p=0·078).

Mortality was strongly associated with age, ranging from 11·1 per 100 CYO (95% CI 7·19–17·30) in children aged 24 months or more, to 31·0 per 100 CYO (95% CI 24·8–39·0) in infants younger than 6 months (p<0·0001 for trend across age groups; appendix).

There were 616 non-fatal admissions to hospital and 3266 non-fatal episodes of illness for which children were treated as outpatients. The incidence of readmission to hospital or death during follow-up was 57·1 per 100 CYO (95% CI 54·6–59·6). We noted no significant differences in the overall rates of hospital admission or outpatient illness between intervention groups (table 3, appendix). The frequency of pneumonia episodes was similar between groups, but diarrhoea occurred more frequently in the group assigned to co-trimoxazole (table 3). Skin or soft tissue infections, culture-confirmed urinary tract infections, and confirmed malaria were less frequent in the group assigned to co-trimoxazole.

**Table 3:** Secondary outcomes

	**Placebo (n=891)**	**Co-trimoxazole (n=887)**	**Incidence rate ratio (95% CI)**
Non-fatal hospital admissions	320	296	0·93 (0·79–1·09)
Hospital admission or death	455	418	0·92 (0·80–1·05)
Outpatient treatment	1666	1600	0·96 (0·89–1·03)
Outpatient, hospital admission, or death	2441	2314	0·95 (0·90–1·00)
All pneumonia	682	633	0·93 (0·83–1·04)
Severe pneumonia	200	193	0·97 (0·79–1·18)
All diarrhoea	458	512	1·14 (1·02–1·28)*
Severe diarrhoea	100	125	1·25 (0·95–1·64)
Confirmed malaria†	42	25	0·60 (0·35–0·99)*
Skin or soft tissue infection	176	136	0·77 (0·61–0·97)*
Urine culture done for clinical indication	80	58	0·73 (0·51–1·03)
Positive urine culture†	41/80 (51·3%)	21/58 (36·2%)	0·51 (0·29–0·89)*
Blood culture done for clinical indication	219	231	1·05 (0·87–1·28)
Positive blood culture†	8/219 (3·7%)	9/231 (3·9%)	1·13 (0·39–3·35)
Started treatment for tuberculosis	49	43	0·88 (0·57–1·35)
Suspected toxicity‡	32	31	0·98 (0·58–1·65)

Data are the number of episodes or n (%) for laboratory tests.

* p<0·05.

† Details presented in the appendix.

‡ While receiving study medication (details in the appendix).

During the trial, of 450 blood cultures done, 17 (3·8%) were positive for likely pathogens (appendix). The overall incidence of detected bacteraemia was 1·13 per 100 CYO (95% CI 0·65–1·78) and the overall incidence of detected culture-positive urinary tract infection was 4·06 per 100 CYO (95% CI 3·11–5·21). Almost all bacterial pathogens isolated from blood and urine cultures were non-susceptible to co-trimoxazole, without evidence of differences between treatment groups.

In the first 6 months of the study (while participants received study medication), 63 suspected grade 3 or 4 adverse events were recorded in 57 (3%) children: 31 (2%) in the co-trimoxazole group and 32 (2%) in the placebo group (incidence rate ratio 0·98, 95% CI 0·58–1·65). The most common adverse events of these grades were urticarial rash (grade 3, equally common in both groups), neutropenia (grade 4, more common in the co-trimoxazole group), and anaemia (both grades equally common in both groups). Scheduled neutrophil counts at months 2 and 6 were lower in the co-trimoxazole group (appendix). One child in the placebo group had fatal toxic epidermal necrolysis with concurrent *Pseudomonas aeruginosa* bacteraemia.

**Figure 3:**
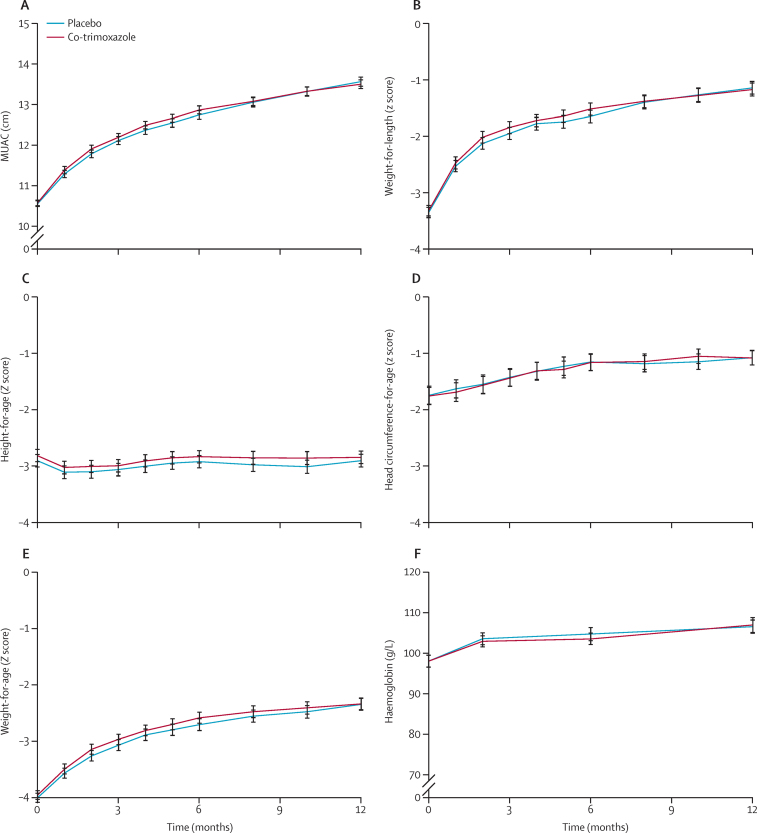
Anthropometry during follow-up Data are means; error bars indicate 95% CI. No individual timepoint comparisons between randomised groups were significant (appendix). MUAC=mid-upper-arm circumference.

For all anthropometric variables assessed, no individual timepoint comparison between intervention groups was significant (figure 3). Mean MUAC, weight-for-height, weight-for-age, and head circumference-for-age increased during follow-up. Mean length-for-age decreased during the first 2 months, indicating a faltering of linear growth, and returned to a value similar to that recorded at enrolment at 6 and 12 months (appendix).

After 12 months, 1209 (68%) of 1778 children enrolled were alive and in follow-up with a MUAC above the threshold for moderate acute malnutrition (≥12·5 cm). However, this outcome varied with age, ranging from 160 (52%) of 306 infants younger than 6 months at enrolment to 154 (78%) of 197 children aged 24 months or more at enrolment, p<0·0001 (appendix).

Overall, 1008 (57%) of 1778 participants were born before 2011 and wouldn't have received PCV as part of their infant immunisation schedule. Of the 770 participants born after introduction of PCV, 630 (82%) had three doses, 116 (15%) had two doses, 85 (11%) had one dose, and 53 (7%) had none. After adjustment for age, we noted no differences in mortality risk between children born before and after 2011: HR 1·03 (95% CI 0·76–1·35). No evidence was apparent for modification of the effect of co-trimoxazole on mortality by birth before or after the introduction of PCV (p=0·512), nor receipt of two or more doses in children born after its introduction (p=0·812).

Diarrhoea and severe pneumonia were the most common reasons for admission to hospital immediately before enrolment, affecting 1499 (84%) of 1778 participants: 541 (30%) with diarrhoea only, 478 (27%) with severe pneumonia only, and 480 (27%) with both these conditions. After adjustment for age, there was evidence of modification of the effect of co-trimoxazole on mortality by these reasons for admission (p=0·036; appendix).

## Discussion

Among Kenyan children with complicated SAM (but without HIV), daily co-trimoxazole given for 6 months was well tolerated, but did not reduce mortality or improve growth. However, as expected, it did result in a lower incidence of malaria and some bacterial infections. The findings from our trial suggest that children with complicated SAM remain susceptible to severe infections and death after discharge from hospital despite provision of recommended medical and nutritional care and enhanced follow-up, with a rate of readmissions to hospital or death of 57 per 100 CYO. Among infants aged 2–11 months, mortality was 22 per 100 CYO, which markedly contrasts with the national mortality estimate, reported in the 2014 Kenya Demographic & Health Survey for the previous 5 years, of 1·7 per 100 CYO among infants aged 1–11 months.^21^

Findings from clinical trials of co-trimoxazole prophylaxis have clearly established benefit for admission to hospital and survival in HIV-infected children.^14^ However, trials with children who have been exposed to HIV but who are not HIV infected, in malaria-endemic areas such as Malawi and Uganda, report protection from malaria with co-trimoxazole prophylaxis, but not from other infections, nor improvements in growth.^22^, ^23^, ^24^ In South Africa, for breastfed children exposed to HIV but not HIV infected in a non-malaria-endemic area, co-trimoxazole did not affect pneumonia occurrence, but diarrhoea increased by a third (results of borderline statistical significance).^25^

During our study, malaria transmission was low in the specific locations that we recruited from on the Kenyan Coast, and either low or absent in Nairobi. However, co-trimoxazole prophylaxis was effective in preventing malaria. Overall, diarrhoea episodes were more frequent in children allocated to co-trimoxazole. Thus, our results are compatible with those from previous trials in children who were exposed to HIV but were not HIV infected.

A key question is whether low bacterial susceptibility to co-trimoxazole in our study contributed to an absence of protective effect on death and the syndromes most commonly associated with serious illness. If so, this result would be of concern for the use of co-trimoxazole prophylaxis in HIV-infected children. In developing countries, the effect of co-trimoxazole appears to be against a range of common infections rather than specifically against *Pneumocystis jirovecii*. Findings from trials in individuals with HIV show similar efficacy of co-trimoxazole prophylaxis in countries with high (>80% in South Africa, Uganda, and Zambia) and low (<33% in Côte d'Ivoire)^20^ prevalence of co-trimoxazole non-susceptibility in invasive bacterial isolates in vitro, using criteria based on treating established infections at standard antimicrobial doses. Additionally, in settings similar to Kenya, such as Uganda and Zimbabwe, co-trimoxazole prevented life-threatening infections in HIV-infected children.^13^

In our trial, we showed that, although the bacteria isolated from blood or urine were largely non-susceptible to co-trimoxazole, the drug did prevent two conditions that are almost always bacterial: urinary tract infection, for which antimicrobial concentrations are higher than in plasma, mainly caused by non-susceptible *Enterobacteriaceae*; and skin and soft tissue infections that were likely to have been caused by *Staphylococcus aureus* and group A streptococci. Considered together, the findings from trials on children with HIV, on children exposed to HIV but not HIV infected, and our trial suggest that the absence of efficacy in our trial compared with those of children with HIV is likely to have been due to fundamental differences in the immunopathology of malnutrition and HIV infection, their associated infections, and their interaction with antimicrobials.

Few positive blood cultures were noted during our trial, despite high numbers of death and readmission, active screening for invasive bacterial disease, and a high prevalence of antimicrobial resistance. The population-based incidence of bacteraemia was not markedly greater than that previously estimated within the general community of this age group on the Kenyan coast.^26^ Although our trial and previous studies might have underestimated bacteraemia because of previous antibiotic use and non-presentation to hospital,^11^ our results raise the possibility that a substantial proportion of serious infections might not have been bacterial. Although detected invasive bacterial infection is generally associated with mortality, it typically accounts for few deaths in similar settings, especially after the introduction of conjugate vaccines against previously major causes of pneumonia and sepsis.^27^, ^28^, ^29^

We chose to investigate the efficacy of co-trimoxazole because of its well documented effect on mortality in children with HIV who present with infectious syndromes that are broadly similar to those noted in children with SAM. Among children with SAM, we noted two main reasons for initial admission: diarrhoea and pneumonia. In an exploratory analysis, we identified evidence that suggested that the reason for admission modified the effect of co-trimoxazole on mortality. This finding raises a hypothesis that SAM with diarrhoea might represent a phenotype amenable to antimicrobial prophylaxis targeting pathogens and commensal microbes, intestinal barrier function, and immune homoeostasis that could be tested in further trials.

Interest arose in the prophylactic use of azithromycin in children after a large, cluster-randomised clinical trial of mass administration in the general population for trachoma control in Ethiopia.^30^ In that trial, childhood mortality was 49% lower in the intervention groups, although the precise mechanism was unknown. In other contexts, azithromycin might prevent malaria, intestinal infections, and infections of the lower-respiratory tract in children.^31^, ^32^, ^33^ Azithromycin, like co-trimoxazole, has some non-specific anti-inflammatory effects and has a good safety profile. Up to now, no trials with high-mortality risk groups such as SAM have been done and azithromycin might be worth testing in these groups. Furthermore, in a prespecified analysis, we observed weak evidence suggesting a protective effect after the 6-month period of allocation to co-trimoxazole. In a trial with children with uncomplicated SAM in Malawi,^2^ differential mortality was noted after the period of antimicrobial administration between groups receiving amoxicillin, cefdinir, or placebo. However, no deaths occurring during the 7 days of treatment in any of the randomised groups. Thus, a shorter period of prophylaxis or longer follow-up might have altered the results of our trial.

There was a high prevalence of severe stunting among participants (69%), as reported previously in SAM.^8^ Stunting was not reversed by therapeutic feeding and medical care during the trial. Thus, although children were recruited in hospital with criteria for SAM, indicators of chronic poor health were usually also present. Underlying factors probably included specific nutritional deficiencies not adequately corrected by therapeutic and supplementary feeding strategies; intestinal inflammation and dysbiosis;^34^ a high burden of exposure to new infections; and social disadvantage and other factors associated with poverty.

Infants younger than 6 months had an especially high mortality (31 per 100 CYO) and poor long-term outcome—only around half this age group had adequate MUAC (≥12·5 cm) after 12 months. Until 2011, SAM was assumed to be uncommon in infants younger than 6 months^35^ or simply a sequela of low birthweight not requiring specific intervention strategies. Consequently, infants younger than 6 months are usually excluded from anthropometric screening for SAM in hospitals, in primary care, and in community nutritional surveys. Diagnostic cutoffs for MUAC have not been internationally agreed. However, in this trial, infants younger than 6 months, recruited using MUAC values, comprised 17% of the participants and 29% of the deaths. An update of WHO guidelines includes treatment recommendations for this age group, but evidence for their efficacy is needed.^4^

Some limitations of our trial were that we were unable to identify whether prescribed therapeutic and supplementary feeding met individual children's nutritional requirements, or to accurately monitor compliance with feeding recommendations at home. We used routine diagnostic microbiology to diagnose infections, rather than enhanced molecular methods that might have improved the diagnosis of those that might have been influenced by co-trimoxazole, including tuberculosis.

Despite an overall global reduction in child mortality, complicated SAM remains common in much of the developing world and is associated with high levels of short-term and long-term mortality. Tackling SAM will require better understanding of the causes of infections, determinants of susceptibility to infections, specific nutritional and metabolic disturbances in relation to therapeutic and supplementary feeding strategies, and the parts played by social and environmental factors.
