# Supplementary material for: Daily co-trimoxazole prophylaxis to prevent mortality in children with complicated severe acute malnutrition: a multicentre, double-blind, randomised placebo-controlled trial
Source: Lancet Glob Health. 2016 Jun 2;4(7):e464–73. doi: 10.1016/S2214-109X(16)30096-1 (PMC6132285; doi:10.1016/S2214-109X(16)30096-1)
Supplement: Supplementary appendix [file mmc1.pdf]

THE LANCET Global Health

Supplementary appendix

This appendix formed part of the original submission and has been peer reviewed. We post it as supplied by the authors.

Supplement to: Berkley JA, Ngari M, Thitiri J, et al. Daily co-trimoxazole prophylaxis to prevent mortality in children with complicated severe acute malnutrition: a multicentre, double-blind, randomised placebo-controlled trial. *Lancet Glob Health* 2016; published online June 2. http://dx.doi.org/10.1016/S2214-109X(16)30096-1.

SUPPLEMENTARY APPENDIX

Daily co-trimoxazole prophylaxis to prevent mortality in children with complicated severe acute malnutrition: a multicentre, double-blind, randomised placebo-controlled trial

James A Berkley, Moses Ngari, Johnstone Thitiri, Laura Mwalekwa, Molline Timbwa, Fauzat Hamid, Rehema Ali, Jimmy Shangala, Neema Mturi, Kelsey DJ Jones, Hassan Alphan, Beatrice Mutai, Victor Bandika, Twahir Hemed, Ken Awuondo, Susan Morpeth, Samuel Kariuki, Gregory Fegan

Correspondence to:
Prof James A Berkley
Medical Research Institute/Wellcome Trust Research Programme
Kilifi 80108, Kenya
jberkley@kemri-wellcome.org

Table of Contents

SUPPLEMENTARY METHODS 3

Study Population 3

Screening and Eligibility 3

Investigational Product 4

Co-trimoxazole Dosage 4

Care and Follow Up 4

Additional Clinical Definitions 5

Laboratory Methods 5

Interim Analyses 5

SUPPLEMENTARY TABLES 6

Table S1: Enrolment by site 6

Table S2: Characteristics of children who survived and died 7

Table S3 Mortality in pre-specified subgroups according to intervention group 8

Table S4. Details of 'Other Infections' causing death, according to intervention group 9

Table S5. Mortality, according to age group 9

Table S6. Non-fatal hospital admissions, according to intervention group 10

Table S7. Clinical events treated as an outpatient, according to intervention group 11

Table S8. Pathogens detected in blood or urine, according to intervention group 12

Table S9. Definitions of grade 3 and 4 toxicity events, according to intervention group 13

Table S10: Grade 3 and 4 toxicity events, according to intervention group 13

Table S11. Blood counts at scheduled assessments, according to intervention group 14

Table S12. Anthropometry at 0, 2, 6 and 12 months, according to intervention group 14

Table S13. Nutritional status after 12 months, according to intervention group 15

Table S14. Nutritional status after 12 months, according to age group 15

Table S15. Effect modification by clinical syndrome causing index admission (post hoc) 16

SUPPLEMENTARY FIGURES 17

Figure S1. Kwashiorkor (oedematous malnutrition), according to age 17

Figure S2: Compliance with study medication, by month 18

Figure S3. Enrolment and mortality, according to age 18

Figure S4. Cumulative hazard of death, according to age group 19

REFERENCES FOR SUPPLEMENTARY APPENDIX 20

TRIAL PROTOCOL 21

SUPPLEMENTARY METHODS

Study Population

We enrolled children at two rural and two urban hospitals in Kenya (Table S1). Kilifi County Hospital (KCH) is located in a rural town on the Indian Ocean coast. The majority of the population in the catchment area of KCH are rural farmers. The KEMRI/Wellcome Trust Research Programme (KWTRP) is located at KCH. Because of another ongoing study of effectiveness of pneumococcal conjugate vaccine, participants who were resident in the Kilifi Health and Demographic Surveillance System (KDHSS) area[1] were not enrolled (approximately 50% of hospital admissions with SAM). Participants largely represented residents of the semi-arid areas inland (West) of the KDHSS. Malindi sub-County Hospital is located within Kilifi County, approximately 60km North of Kilifi, also on the coast. It serves a mainly rural population living in a semi-arid area to the West and North to the border with Lamu District, and Malindi Town. Coast General Hospital (CGH), Mombasa is the largest provincial hospital in Kenya. CGH predominantly receives admission from Mombasa Town and referrals from the entire coastal area. Mombasa includes several low-income informal settlement areas (slums). Mbagathi Hospital, Nairobi is located centrally in Nairobi, near the Kibera slum and mostly serves populations living in low-income informal settlements throughout Nairobi. An estimated sixty percent of the city's population live in slums, which occupy less than 6 percent of the residential land in Nairobi.

Screening and Eligibility

Screening of all paediatric admissions for eligibility was conducted at, or soon after admission to hospital. All children admitted to the study hospitals were weighed using an electronic scale (Seca 825, Birmingham, UK) to 0.01 kg. Length was measured to 0.1 cm using an infantometer (Seca 416, Birmingham, UK) or stadiometer (Seca 215, Birmingham, UK). Mid-upper arm circumference (MUAC) was measured to 0.1 cm using a standard insertion tape (TALC, St. Albans, UK); and kwashiorkor was diagnosed on the basis of bilateral pitting pedal oedema.[2]

MUAC criteria for eligibility were initially <11.0cm for children aged 6 months or more and age-stratified cut-offs between 10.5 and 11.0cm for infants aged 2 to 6 months (see Supplementary Appendix). In April 2011, MUAC criteria were amended in order to reflect changes in national guidelines and simplify enrolment to <11.5cm for children aged 6 months or more and <11.0cm for infants aged 2 to 6 months. Exclusion criteria were known allergy to co-trimoxazole; serious comorbidity likely to be associated with mortality unrelated to infection such as severe heart disease or malignancy; and residence outside the attending hospital's catchment area.

Children were enrolled once they had completed the stabilization phase and were in the rehabilitation phase for SAM of treatment according to:

- *Absence of any WHO danger or emergency signs: obstructed breathing, respiratory distress, cyanosis, shock (delayed capillary refill plus fast & weak pulse plus temperature gradient), severe anaemia (Hb <5g/dl), congestive cardiac failure, impaired consciousness, convulsions, severe dehydration, profuse watery diarrhoea, vomits everything, hypothermia;*
- <u>*and*</u> *if there was oedema at baseline, loss of oedema defined as improving from a severe +++ oedema (severe: generalized bilateral pitting oedema including feet, legs, arms and face) to ++ oedema (moderate:*

no upper arm or upper leg oedema and no facial oedema or from ++ oedema to + (mild: only feet/ankle oedema) or none;

- *<u>and</u> tolerating full prescribed volume of F75 feeds and observed to be completing the feeds.*

HIV antibody testing was offered to all children on a provider-initiated testing and counselling (PITC) basis, according to national guidelines. Although within the inclusion criteria, there was provision to enrol children with a positive HIV antibody test but negative confirmatory PCR (HIV exposure only), in practice, it was not possible to obtain PCR results in time, and only children with a negative HIV antibody test were enrolled.

Investigational Product

Trial co-trimoxazole and placebo were manufactured by Cosmos Pharmaceuticals, Nairobi and supplied in sealed blister packs. The investigational products were tested for drug content, disintegration and uniformity of weight at September 2009 and again in February 2013 by the National Quality Control Laboratory, Nairobi. They were distributed in sealed blister packs in small boxes holding one month's supply, marked with the child's initials and study number. The caretaker was also given a plastic cup to use to dissolve the tablets. After randomization and distribution of the study interventions, the study team educated each caretaker on how to dissolve dispersible tablets in clean water and give the medication. The administration of study medication was by caretakers and observed whilst the child remained in hospital.

Co-trimoxazole Dosage

Dosage for the trial was according to recommendations for prophylaxis amongst children with HIV.[3] The intervention group were prescribed 120mg (20mg trimethoprim/100mg sulphamethoxazole) for infants under 6 months old, and 240mg (40mg trimethoprim/200mg sulphamethoxazole) for children age 6 months or more. The dose for infants under 6 months at enrolment was increased from 120 to 240mg once they reached 6 months of age. By bodyweight, the mean prescribed daily dose, expressed as total (trimethoprim/ sulphamethoxazole) was 39 (6.5/32.5)mg/kg sd (9.1) at enrolment and 31 (5.2/25.8)mg/kg sd (7.2) after 6 months. For comparison, in the recently published ARROW trial in Uganda and Zimbabwe,[10,11] a dose of 240mg was used for children weighing 5 to 15Kg, giving a range of dosage between 16 (13/3)mg/kg to 48 (40/8)mg/kg.

Care and Follow Up

Training was given to trial clinicians and ward assistants on anthropometry, GCP, and research ethics. Trial clinicians and ward staff were trained in the recognition and management of severely ill children and in the treatment and care of SAM.

Children were treated according to WHO guidelines (2005)[2] for the first phase of treatment of complicated SAM, including F75 milk feeds and broad spectrum intravenous antibiotics. Information was given about the trial to parents or carers of potentially eligible children and they were given the opportunity to ask questions and discuss with fathers and other relatives. Following confirmation of eligibility and written informed consent, enrolment occurred once children had stabilized and begun taking F100 milk feeds. Therapeutic feeding for infants under 6 months old was done using expressed breast milk or dilute F100 for infants by cup and spoon. Advice on breastfeeding and complementary feeding was given during the admission.

A mobile phone 'hotline' was provided for participants to seek advice or inform the study team of clinical events. Free walk-in clinic services were offered for unscheduled visits at the trial follow-up clinic and these illness events were documented. In the event of re-admission to hospital being required, the trial provided costs of fares, bed-charges, drugs and investigations. At each follow up visit, anthropometry was performed as above and adherence assessed by pill counting (caretakers were asked to retain and bring empty blister strips and packets). Details of any admissions to non-study hospitals were sought. Participants travelling temporarily were given a supply of study medication and vital status was confirmed by cell phone at scheduled follow up dates. Participants were considered lost to follow up if they were untraceable two months after a scheduled follow up visit.

Trial clinicians were trained in how to recognize common adverse reactions to co-trimoxazole such as rash, neutropenia, anaemia and jaundice, and what to do if they occurred (see definitions in Table S3). Complete blood counts were measured at baseline and months 2, 6 and 12. Blood counts and other laboratory investigations relating to potential toxicities were undertaken if clinically indicated.

Additional Clinical Definitions

Sepsis was clinically defined within the capacity of the participating hospitals, based on a suspected or confirmed source of infection, hyper-or hypothermia and tachycardia, but not abnormal leucocyte counts.

Urinary tract infection was defined as urinary tract infection defined as one or more of fever, urinary frequency or dysuria plus either leucocyte esterase or nitrite on urine dipstick testing. Confirmed urinary tract infection was defined as these plus a positive urine culture.

Laboratory Methods

Full blood counts were performed by Coulter counter (Beckman Coulter, High Wycombe, UK). Blood cultures were collected and processed in BACTEC Peds Plus bottles with a BACTEC 9050 instrument (Becton Dickinson, Oxford, UK). Positive samples were sub-cultured on standard media by routine microbiological techniques. Urinalysis was done by reagent dipsticks (Mission, Acon Laboratories Inc., San Diego, USA). If the urine was positive for leucocytes or nitrites, a sample was sent for culture. Urine was cultured on Cysteine-Lactose-Electrolyte Deficient agar at 37°C. A positive culture was defined as growth of a single urinary tract pathogen at ≥50 CFU/µl. Bacterial susceptibility was tested by disk diffusion (British Society for Antimicrobial Chemotherapy). Malaria testing was done by Giemsa stained blood slide examined at x1000 in Kilifi, according to standard methods; and by rapid diagnostic test (Optimal, Flow Inc., Oregon, USA), which detects a malaria parasite lactate dehydrogenase, at the other sites.

Interim Analyses

Five interim analyses by treatment group were conducted by the independent DSMC in closed session, but not disclosed to the study team. Stopping rules in case of evidence of benefit or harm prior to completion of the trial were guided by the Haybittle-Peto boundary, a probability of <0.001. The final analyses were evaluated at a two-sided alpha level of 0.05.

SUPPLEMENTARY TABLES

Table S1: Enrolment by site

	Started Enrolling	Ended Enrolling	Last Follow up	Number Enrolled
Coast General Hospital, Mombasa (urban)	20/11/2009	19/02/2013	20/01/2014	849
Kilifi County Hospital, Kilifi (rural)	22/02/2010	31/03/2013	09/04/2014	151
Malindi sub-County Hospital, Kilifi (rural)	25/03/2010	21/03/2013	24/03/2014	271
Mbagathi Hospital, Nairobi (urban)	10/05/2011	29/03/2013	28/03/2014	507

Data are dates or n.
Children enrolled at rural sites were older than those enrolled at urban sites: median age 13 months; IQR 7 to 21 months versus 10 months; IQR 6 to 15 months (Wilcoxon rank sum test P=0.001).

Table S2: Characteristics of children who survived and died

	Death during index admission (N=60)	Deaths after discharge within 6 months (N=150)	Deaths after 6 months (N=47)	Survivors (N=1,521)
Age — months median (IQR)	11.8 (8.5-18.9)	7.3 (4.7-11.7)	7.1 (5.3-13.8)	11.4 (7.6-17.0)
Age less than 6 months	9 (15)	52 (35)	15 (32)	230 (15)
Gender (female)	27 (45)	80 (53)	23 (49)	745 (49)
Mother is primary caretaker	54 (90)	139 (93)	43 (91)	1425 (94)
Caretaker completed primary education n/total (%) ‡	8 (13.3)	50 (33)	16 (34)	632 (42)
Current breast-feeding	36 (60)	109 (73)	31 (66)	916 (60)
Received conjugate pneumococcal vaccine §	23 (38)	85 (57)	27 (57)	893 (59)
Urban site	41 (68)	127 (85)	33 (70)	1155 (76)
Nutritional oedema	15 (25)	11 (7.3)	4 (8.5)	270 (18)
MUAC — cm	9.9 ±1.34	10.2 ±1.24	10.1 ±0.95	10.6 ±1.04
MUAC-for-age z score ¶	-4.5 ±1.19	-3.9 ±0.98	-4.2 ±0.96	-3.8 ±1.01
Weight-for-length z score ǁ	-4.0 ±1.51	-3.3 ±1.29	-3.3 ±1.31	-3.3 ±1.24
Weight-for-age z score ǁ	-4.9 ±1.20	-4.1 ±1.20	-4.3 ±1.11	-3.9 ±1.07
Length-for-age z score	-3.6 ±2.02	-2.8 ±1.80	-3.2 ±1.88	-2.8 ±1.61
Head circumference-for-age z score ‡	-2.3 ±1.72	-1.8 ±1.48	-2.2 ±2.10	-1.7 ±1.40
Haemoglobin g/dl	8.99 ±2.24	9.94 ±1.93	9.66 ±2.19	9.82 ±2.21
Clinical signs of rickets	13 (22%)	24 (16)	11 (23)	194 (13%)
Eye signs of vitamin A deficiency	0	0	0	4 (0.26)
Cerebral palsy	1 (1.67)	10 (6.7)	5 (11)	50 (3.3)
Known tuberculosis at enrolment	1 (1.67)	10 (6.7)	2 (4.3)	54 (3.6)
Index admission for pneumonia	26 (43)	111 (74)	32 (68)	789 (52)
Index admission for diarrhoea	37 (62)	67 (45)	24 (51)	893 (591)
Treated for shock before enrolment	10 (17)	8 (5.3)	2 (4.3)	164 (11)
Impaired consciousness before enrolment	4 (6.7)	15 (10)	4 (8.5)	90 (5.9)
Days from admission to enrolment — median (IQR)	5 (4-7)	7 (5-11)	6 (6-10)	6 (4-8)
Allocated to co-trimoxazole	30 (50%)	76 (50%)	17 (36%)	765 (50%)

Data are n (%), mean ± sd, or median (IQR).
† Plus–minus values are means ± SD.
‡ Data collected from April 2011
§ Received at least one dose of conjugate pneumococcal vaccine at enrolment
¶ Excludes infants under 3 months old, as there is no WHO (2006) reference below this age
ǁ Excludes children with kwashiorkor (oedematous malnutrition)

Table S3 Mortality in pre-specified subgroups according to intervention group

Age Group	Placebo N=891	Co-trimoxazole N=887	Hazard Ratio (95% CI)
Age 2 to 5 months	43/158	32/148	0.79 (0.50 to 1.25)
Age 6 to 11 months	55/343	50/340	0.91 (0.62 to 1.34)
Age 12 to 23 months	29/295	28/297	0.95 (0.57 to 1.61)
Age 24 to 59 months	8/95	12/102	1.42 (0.58 to 3.48)
Log-likelihood ratio test for heterogeneity of effect P=0.715			
**Kwashiorkor**			
Present	14/149	16/151	1.14 (0.55 to 2.33)
Absent	121/742	106/736	0.88 (0.68 to 1.14)
Log-likelihood ratio test for heterogeneity of effect P=0.511			
**Site**			
Coast General Hospital, Mombasa	72/424	64/425	0.87 (0.62 to 1.22)
Kilifi County Hospital, Kilifi	8/73	4/78	0.44 (0.13 to 1.47)
Malindi sub-County Hospital, Kilifi	24/138	20/133	0.88 (0.48 to 1.58)
Mbagathi Hospital, Nairobi	31/256	34/251	1.15 (0.71 to 1.87)
Log-likelihood ratio test for heterogeneity of effect P=0.482			
**Study Period**			
First 6 months (receiving study drug)	105/891	105/887	1.00 (0.77 to 1.32)
Second 6 months (not receiving study drug)	30/758	17/752	0.56 (0.31 to 1.02)
Log-likelihood ratio test for heterogeneity of effect P=0.078			

Data are n/total
The overall incidence of death was 16.8 (95% CI 14.9 to 19.0) per 100 cyo. During the first 6 months of follow up, the incidence of death was 26.4 (95% CI 23.4 to 29.7) per cyo.

Table S4. Details of 'Other Infections' causing death, according to intervention group

	Placebo	Co-trimoxazole
Meningitis	2	3
Confirmed Falciparum malaria†	2	0
Unconfirmed Falciparum malaria‡	4	1
Cerebral abscess§	1	0
Other encephalopathies	8	7
Tuberculosis	2	3
Pericardial effusion	1	2
Febrile illness, cause unknown, death in a study hospital	3	1
Febrile illness , cause unknown, death at a non-study hospital	7	2
Febrile illness , cause unknown, death in the community	17	8
**Total**	**47**	**27**

Data are n.
† Confirmed by blood slide or rapid diagnostic test at one of the study hospitals
‡ Diagnosed at a non-study health facility
§ Diagnosed at post mortem

Table S5. Mortality, according to age group

Age group	N	Deaths	% died*	Incidence Rate per 100 child-years (95% CI)
Age 2 to 5 months	306	75	24.5	31.0 (24.8 to 39.0)**
Age 6 to 11 months	683	105	15.4	18.2 (15.0 to 22.0)**
Age 12 to 23 months	592	57	9.6	10.8 (8.33 to 14.0)
Age 24 to 59 months	197	20	10.1	11.1 (7.19 to 17.3)
Total	1,778	257	14.5	16.8 (14.9 to 19.0)

Data are n or IRR (95% CI)
* Non-parametric test for trend across age groups P<0.001
** Age 2 to 11 months 22.0 (19.0 to 25.4) per 100 CYO

Table S6. Non-fatal hospital admissions, according to intervention group

	Placebo	Co-trimoxazole	Incidence Rate Ratio (95% CI)
Non-fatal hospitalization episodes during 12 months †	320	296	0.92 (0.79 to 1.09)
First 6 months (receiving study drug)	242	232	0.96 (0.80 to 1.15)
Second 6 months (not receiving study drug)	78	64	0.81 (0.57 to 1.14)
Log-likelihood ratio test for heterogeneity of effect P=0.356			
Severe pneumonia during 12 months	154	144	0.93 (0.74 to 1.17)
First 6 months (receiving study drug)	123	116	0.94 (0.73 to 1.23)
Second 6 months (not receiving study drug)	31	28	0.89 (0.51 to 1.53)
Log-likelihood ratio test for heterogeneity of effect P=0.843			
Diarrhoea during 12 months	75	98	1.30 (0.95 to 1.77)
First 6 months (receiving study drug)	61	74	1.21 (0.85 to 1.73)
Second 6 months (not receiving study drug)	14	24	1.69 (0.84 to 3.54)
Log-likelihood ratio test for heterogeneity of effect P=0.380			
Sepsis during 12 months	8	8	1.00 (0.38 to 2.67)
First 6 months (receiving study drug)	7	7	1.00 (0.35 to 2.86)
Second 6 months (not receiving study drug)	1	1	0.99 (0.06 to 15.8)
Log-likelihood ratio test for heterogeneity of effect P=0.991			
Confirmed malaria during 12 months	13	7	0.53 (0.18 to 1.44)
First 6 months (receiving study drug)	9	5	0.56 (0.15 to 1.84)
Second 6 months (not receiving study drug) 0	4	2	0.49 (0.04 to 3.44)
Log-likelihood ratio test for heterogeneity of effect P=0.908			
Other infections during 12 months ‡	100	101	1.00 (0.75 to 1.33)
First 6 months (receiving study drug)	78	82	1.05 (0.77 to 1.45)
Second 6 months (not receiving study drug)	22	19	0.85 (0.46 to 1.58)
Log-likelihood ratio test for heterogeneity of effect P=0.135			
Non-febrile admissions during 12 months §	57	33	0.58 (0.36 to 0.90)*
First 6 months (receiving study drug)	39	26	0.67 (0.39 to 1.12)
Second 6 months (not receiving study drug)	18	7	0.38 (0.13 to 0.96)*
Log-likelihood ratio test for heterogeneity of effect P=0.452			

Data are n or IRR (95% CI)

* P<0.05

† The table includes 54 episodes where children were clinically judged to require admission but parents declined immediate admission, and 91 inpatient deteriorations that prolonged hospital stay.

‡ includes hospitalizations due to meningitis or encephalitis, tuberculosis, measles, abscess, infectious hepatitis and undetermined febrile illnesses without pneumonia, diarrhoea or sepsis.

§ including severe anaemia, cerebral palsy, epilepsy, burns, sickle cell non-febrile crisis, new episode of complicated severe malnutrition with failed appetite test but without fever.

Table S7. Clinical events treated as an outpatient, according to intervention group

	Placebo	Co-trimoxazole	Incidence Rate Ratio (95% CI)
All outpatient episodes during 12 months	1666	1600	0.96 (0.89 to 1.03)
First 6 months (receiving study drug)	1103	1043	0.95 (0.88 to 1.04)
Second 6 months (not receiving study drug)	563	557	0.98 (0.87 to 1.10)
Log-likelihood ratio test for heterogeneity of effect P=0.713			
URTI during 12 months	586	599	1.02 (0.91 to 1.14)
First 6 months (whilst receiving study drug)	401	397	0.98 (0.86 to 1.14)
Second 6 months (after receiving study drug)	185	202	1.08 (0.88 to 1.32)
Log-likelihood ratio test for heterogeneity of effect P=0.519			
LRTI during 12 months	482	440	0.91 (0.80 to 1.03)
First 6 months (whilst receiving study drug)	336	303	0.90 (0.77 to 1.05)
Second 6 months (after receiving study drug)	146	137	0.93 (0.73 to 1.17)
Log-likelihood ratio test for heterogeneity of effect P=0.882			
Diarrhoea during 12 months	358	387	1.08 (0.93 to 1.24)
First 6 months (whilst receiving study drug)	210	234	1.11 (0.92 to 1.34)
Second 6 months (after receiving study drug)	148	153	1.02 (0.82 to 1.28)
Log-likelihood ratio test for heterogeneity of effect P=0.532			
SSTI during 12 months	171	132	0.77 (0.61 to 0.97)*
First 6 months (whilst receiving study drug)	105	70	0.67 (0.49 to 0.92)**
Second 6 months (after receiving study drug)	66	62	0.95 (0.66 1.36)
Log-likelihood ratio test for heterogeneity of effect P=0.167			
UTI during 12 months	39	31	0.79 (0.48 to 1.31)
First 6 months (whilst receiving study drug)	27	15	0.56 (0.28 to 1.09)
Second 6 months (after receiving study drug)	12	16	1.32 (0.62 to 2.78)
Log-likelihood ratio test for heterogeneity of effect P=0.083			
Malaria during 12 months	26	18	0.69 (0.38 to 1.26)
First 6 months (whilst receiving study drug)	13	9	0.70 (0.30 to 1.63)
Second 6 months (after receiving study drug)	13	9	0.69 (0.46 to 1.89)
Log-likelihood ratio test for heterogeneity of effect P=0.974			

Data are n or IRR (95% CI)
*P<0.05, **P<0.001
UTRI = upper respiratory tract infection
LRTI = syndrome of non-severe pneumonia defined as per WHO 2005.[2]
Diarrhoea = three or more loose stools per 24 hours.[2]
SSTI = skin or soft tissue infection including furunculosis, boils, cellulitis, pustules, orchitis, balanitis, scabies.
UTI = urinary tract infection defined as one or more of fever, urinary frequency or dysuria plus either leucocyte esterase or nitrite on urine dipstick testing.
Malaria= Plasmodium falciparum confirmed by blood slide or rapid diagnostic test

Table S8. Pathogens detected in blood or urine, according to intervention group

**Blood Culture** †	**Placebo**	**Co-trimoxazole**	**Incidence Rate Ratio (95% CI)**
Blood Cultures Performed	219	231	1.05 (0.87 to 1.28)
Total Positive Blood Cultures	8	9	1.13 (0.39 to 3.35)
Non-typhoidal *Salmonella*	2	4	2.00 (0.29 to 22.1)
Streptococcus pneumoniae	3	0	-
Staphylococcus aureus	0	2	-
Beta haemolytic streptococcus	1	0	-
Escherichia coli	1	0	-
Klebsiella pneumoniae	0	1	-
Pseudomonas aeruginosa †	1	0	-
Acinetobacter sp.	0	1	-
Enterococcus sp. †	0	1	-
Organism non-susceptible to co-trimoxazole ‡	7/8 (88%)§	8/8 (100%)	-
**Urine Culture** †			
Urine Cultures Performed	80	58	0.73 (0.51 to 1.03)
Total Positive Urine Cultures	41	21	0.51 (0.29 to 0.89)*
Escherichia coli	17	13	0.76 (0.34 to 1.67)
Klebsiella sp.	17	5	0.29 (0.08 to 0.83)*
Escherichia coli and Klebsiella sp.	1	0	-
Enterobacter sp.	2	1	0.50 (0.01 to 9.60)
Enterococcus sp.	2	0	-
Proteus sp.	0	1	-
Staphylococcus aureus	1	0	-
Beta haemolytic streptococcus	0	1	-
Candida albicans	1	0	-
Organism non-susceptible to co-trimoxazole ‡	34/35 (97%)§	17/17 (100%)	-
**Malaria slide or Rapid Diagnostic Test**†			
Positive Malaria Tests	42	25	0.60 (0.35 to 0.99)*

Data are n or IRR (95% CI)
* P<0.05
† All pathogens detected during fatal and non-fatal hospital admissions and outpatient clinical events are presented together.
‡ Enterococcus, Pseudomonas and Candida sp.: these organisms were not tested for susceptibility to co-trimoxazole but were assumed to be non-susceptible.

§ One isolate from blood culture and one from urine were lost and could not therefore be tested.
Systematic blood cultures had also been performed at admission prior to enrolment at one site: Kilifi County Hospital. Of 151 participants enrolled, 7 had bacteraemia detected on admission (4.6%; 95% CI, 1.9 to 9.3): 2 with *Streptococcus pneumoniae*; 1 with *Streptococcus pneumoniae* and non-typhoidal Salmonella; and 1 with each of non-typhoidal Salmonella, *Escherichia coli*, *Klebsiella pneumoniae* and *Staphylococcus aureus*.

Three children had consecutive positive urine cultures: two children had positive cultures approximately 2 weeks apart (one child with *E. coli* on both occasions and one child with Klebsiella sp. on both occasions); the third child had 5 consecutive positive cultures during a period of 3 months (2 *E. coli* and 3 Klebsiella sp.). All three children were in the placebo group.

Table S9. Definitions of grade 3 and 4 toxicity events, according to intervention group

Definition	Grade 3	Grade 4
Allergic reaction	Mild to moderate urticaria	Severe urticaria, angiooedema, anaphylaxis or bronchospasm requiring treatment
Cutaneous reactions	Vesiculation or ulceration	Exfoliative dermatitis, including Stevens- Johnson syndrome and toxic epidermal necrolysis
Severe anaemia	Haemoglobin <4g/dl	Haemoglobin <4 g/dl with evidence of shock, respiratory distress or cardiac failure.
Neutropenia	Neutrophil count <0.4 x 10^9 /L	Neutrophil count <0.25 x 10^9 /L
Abnormal liver function tests	Transaminases or gamma-glutamyl transferase 10-15 x upper limit of normal:<br><br>ALT 370-555 IU/L<br>AST 420- 630 IU/L<br>GGT 460-690 IU/L<br><br>Bilirubin 3.0-7.5 x normal: (50-128 µmol/L)	Transaminases or gamma-glutamyl transferase >15 x upper limit of normal:<br><br>ALT >555 IU/L<br>AST >630 IU/L<br>GGT >690 IU/L<br><br>Bilirubin >7.5 x normal: (>128 µmol/L)

Definitions were derived from the WHO Guidelines on Co-trimoxazole Prophylaxis for HIV-related Infections among Children, Adolescents and Adults Recommendations for a Public Health Approach – Annex 5,[3] except for severe anaemia, for which the WHO Pocket Book for Hospital Care of Children: guidelines for the management of common illness with limited resources (2005) was used.[4]

Table S10: Grade 3 and 4 toxicity events, according to intervention group

Type of toxicity†	Placebo<br>**N=891**	Co-trimoxazole<br>**N=887**
All suspected toxicities	32	31
Urticarial rash (grade 3)	17	15
Lip swelling (grade 4)	1	0
Toxic epidermal necrolysis (grade 4)	1	0
Severe anaemia (grade 3)	3	3
Severe anaemia (grade 4)	4	4
Neutropenia (grade 3)	3	1
Neutropenia (grade 4)	1	7*
Abnormal liver function tests (grade 3)	2	1
Abnormal liver function tests (grade 4)	0	0

Data are n or IRR (95% CI)
*P<0.05
† Data are numbers of events that occurred whilst receiving study medication, excluding pre-existing conditions at randomization.
There were 63 separate events occurring in 57 children: incidence rate 7.9 per 100 cyo; 95% CI 6.2 to 10.0, incidence rate ratio between groups 1.04; 95% CI, 0.62 to 1.76.

Table S11. Blood counts at scheduled assessments, according to intervention group

Time	Group	Haemoglobin g/dl	White blood cells x10^9/L	Neutrophils x10^9/L	Lymphocytes x10^9/L	Platelets x10^9/L
**Enrolment**	Placebo	9.8 (8.5-11.0)	9.8 (6.8-13.2)	3.1 (1.9-4.9)	4.9 (3.0-7.2)	402 (237-557)
	Co-trimoxazole	9.8 (8.4-11.0)	9.9 (6.8-13.1)	3.2 (1.9-4.8)	4.9 (2.2-7.0)	400 (239-560)
**Month 2**	Placebo	10.3 (9.2-11.4)	9.9 (7.3-13.2)	3.2 (2.2-4.8)	5.2 (3.2-7.1)	406 (281-535)
	Co-trimoxazole	10.4 (9.2-11.3)	9.6 (6.6-12.5)*	2.8 (1.8-4.3)**	5.0 (2.9-7.1)	419 (263-535)
**Month 6**	Placebo	10.3 (9.4-11.6)	9.8 (7.2-12.8)	3.2 (2.2-4.8)	4.8 (3.3-7.0)	387 (240-513)
	Co-trimoxazole	10.5 (9.2-11.4)	9.6 (7.2-12.2)	3.0 (2.0-4.4)*	5.0 (3.4-6.6)	381 (262-498)
**Month 12**	Placebo	10.7 (9.4-11.8)	9.8 (7.1-12.9)	3.6 (2.3-5.4)	4.8 (3.0-6.7)	367 (252-501)
	Co-trimoxazole	10.7 (9.5-11.8)	9.6 (7.2-12.1)	3.4 (2.3-4.9)	4.7 (3.0-6.2)	363 (216-512)

Data are median (interquartile range)
* P<0.05, ** P<0.001

Table S12. Anthropometry at 0, 2, 6 and 12 months, according to intervention group

	Group	MUAC cm	Weight-for-Length Z Score	Weight-for-Age Z Score	Length-for-Age Z Score	Head Circumference-for-Age Z score
**Enrolment**	Placebo	10.6 (1.09)	-3.35 (1.26)	-4.01 (1.11)	-2.91 (1.67)	-1.82 (1.47)
	Co-trimoxazole	10.6 (1.05)	-3.32 (1.27)	-3.96 (1.10)	-2.82 (1.64)	-1.73 (1.40)
**Month 2**	Placebo	11.8 (1.33)**	-2.13 (1.42)**	-3.27 (1.24)**	-3.10 (1.55)**	-1.60 (1.49)**
	Co-trimoxazole	11.9 (1.26)**	-2.01 (1.46)**	-3.15 (1.25)**	-3.01 (1.50)**	-1.57 (1.43)**
**Month 6**	Placebo	12.7 (1.39)**	-1.65 (1.45)**	-2.71 (1.33)**	-2.93 (1.42)	-1.18 (1.42)**
	Co-trimoxazole	12.9 (1.29)**	-1.52(1.43)**	-2.59 (1.28)**	-2.84 (1.41)	-1.18 (1.43)**
**Month 12**	Placebo	13.6 (1.40)**	-1.14 (1.38)**	-2.35 (1.28)**	-2.91 (1.37)	-1.07 (1.40)**
	Co-trimoxazole	13.5 (1.34)**	-1.17 (1.44)**	-2.34 (1.32)**	-2.84 (1.38)	-1.08 (1.41)**

Data are mean (sd)
All comparisons between treatment allocation groups using unpaired t-tests were P>0.05, therefore these are not individually indicated.
Compared to enrolment values using paired t-tests to evaluate nutritional recovery: * P<0.05, ** P<0.001
Values for all follow up visits are shown in Figure 2 in the main article.

Table S13. Nutritional status after 12 months, according to intervention group

	Placebo N=891		Co-trimoxazole N=887	
	N	%	N	%
MUAC ≥12.5cm	603	67.7	602	67.9
MUAC 11.5 to 12.4cm	73	8.2	82	9.2
MUAC <11.5cm or kwashiorkor	36	4.0	34	3.8
Died	135	15.2	122	13.8
Lost to follow up	44	4.9	47	5.3
Data are n %				Chi squared test P=0.857

Table S14. Nutritional status after 12 months, according to age group

	24 to 59 months N=197		12 to 23 months N=592		6 to 11 months N=683		2 to 5 months N=306	
	N	%	N	%	N	%	N	%
MUAC ≥12.5cm	154	78.2	457	77.2	434	63.5	160	52.3
MUAC 11.5 to 12.4	11	5.6	35	5.9	74	10.8	35	11.4
MUAC <11.5cm or kwashiorkor	7	3.6	13	2.2	27	4.0	23	7.5
Died	20	10.2	57	9.6	105	15.4	75	24.5
Lost to follow up	5	2.5	30	5.1	43	6.3	13	4.2
Data are n %								Chi squared test P<0.001

Table S15. Effect modification by clinical syndrome causing index admission (post hoc)

Syndrome causing admission	Age median months	Deaths in placebo group	Deaths in co-trimoxazole group	Hazard Ratio 95% CI	P
Diarrhoea only	12.1	35/271 (13%)	19/270 (7.0%)	0.54 (0.31 to 0.94)	0.029
Diarrhoea and Pneumonia	8.9	40/235 (17%)	34/245 (14%)	0.68 (0.48 to 0.96)	0.031
Pneumonia only	9.7	48/249 (19%)	47/229 (21%)	1.09 (0.73 to 1.63)	0.667
No diarrhoea or pneumonia	17.9	12/136 (8.8%)	22/143 (15%)	1.81 (0.90 to 3.66)	0.099

Likelihood ratio test for heterogeneity of effect, adjusted for age P=0.034

Data are median or n (%)

SUPPLEMENTARY FIGURES

Figure S1. Kwashiorkor (oedematous malnutrition), according to age

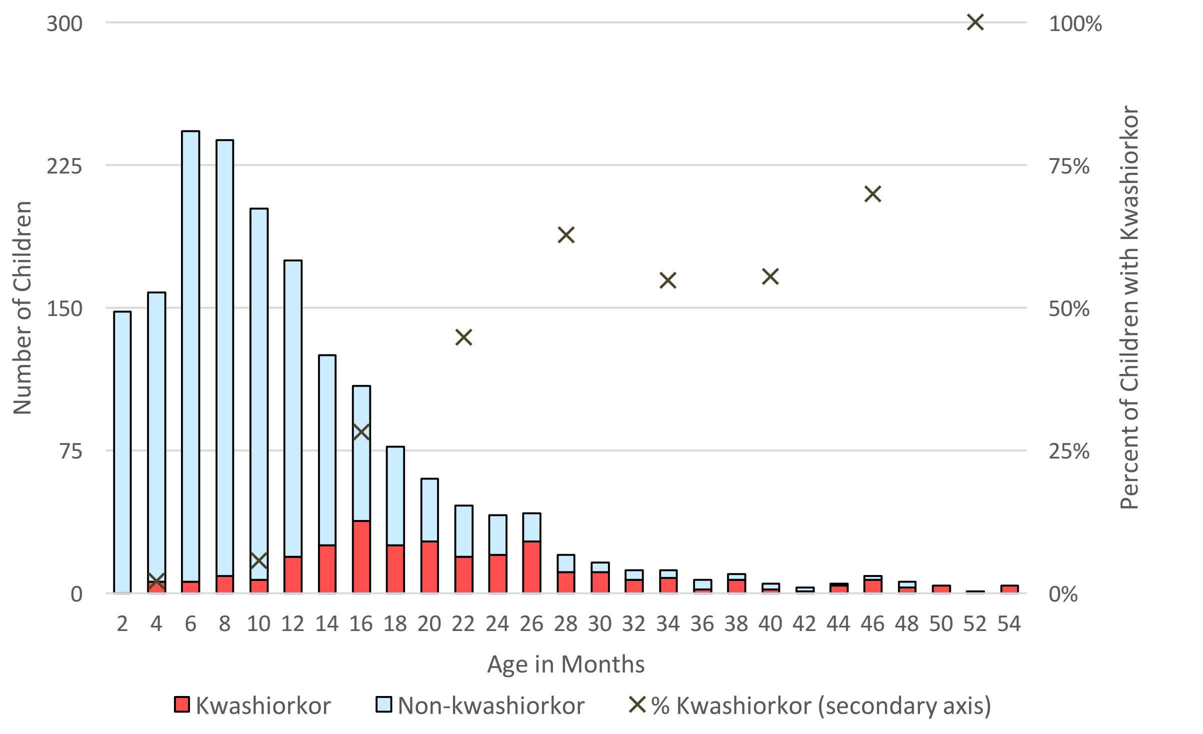

The 300 (16.9%) participants who presented with kwashiorkor (nutritional oedema) at admission were older than those with a low MUAC only: median age 21 months; IQR 16 to 27 months versus 9 months; IQR 6 to 14 months (Wilcoxon rank-sum test, P<0.001). Amongst infants under 6 months, kwashiorkor was present in 2.0% whilst amongst children aged 24 to 59 months, kwashiorkor was present in 60.4% (non-parametric test for trend, P<0.001).

Children with kwashiorkor at enrolment had lower subsequent mortality than amongst children with low MUAC alone: 30/300 (10.0%) compared with 227/1,478 (15.4%) (hazard ratio 0.63; 95% CI 0.43 to 0.92, P=0.017). However, after adjusting for age, there was no evidence for a difference in mortality risk (adjusted hazard ratio 0.91; 95% CI 0.57 to 1.42, P=0.686).

Figure S2: Compliance with study medication, by month

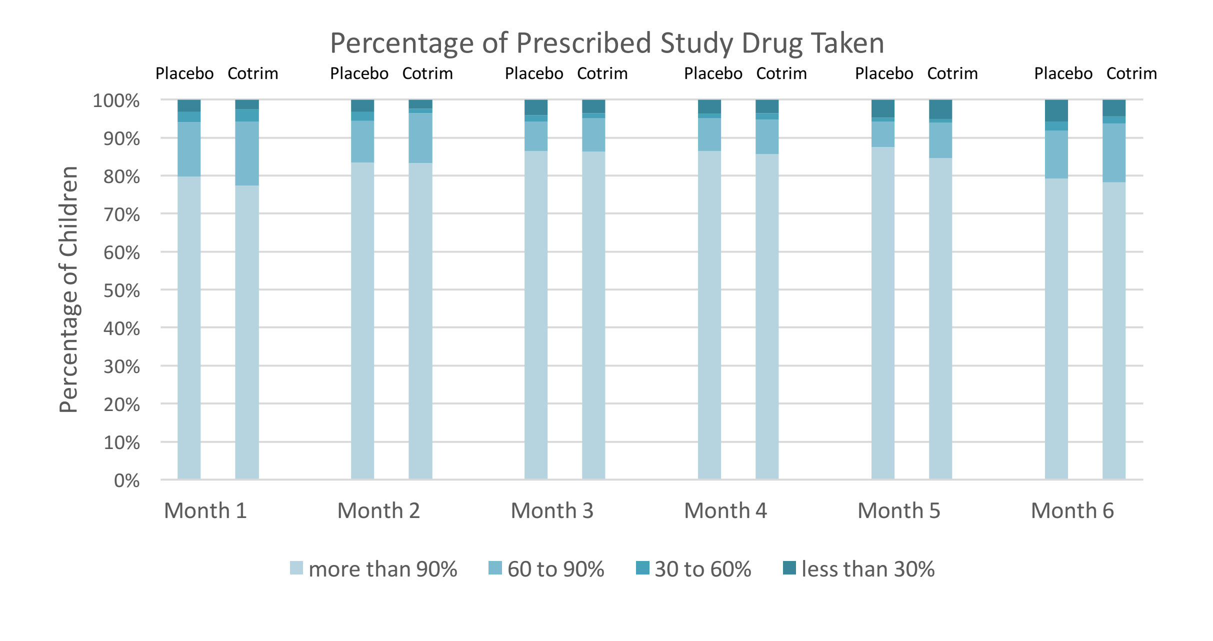

The height of the bars indicates the proportion of participants taking >90%, 60 to 90%, 30 to 60% and <30% of study medication by pill counting and caretaker reports (Wilcoxon rank sum test: P>0.05).

Figure S3. Enrolment and mortality, according to age

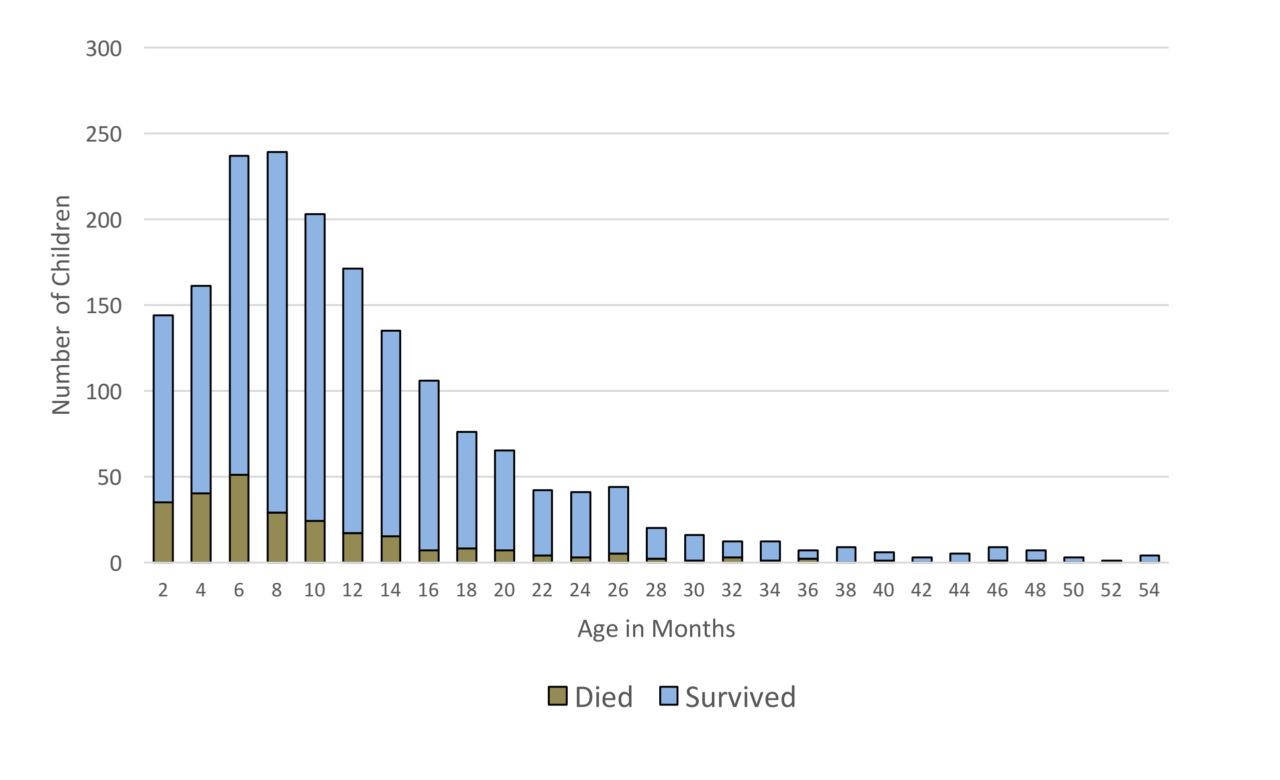

The median age of children who died was 8 months; IQR 6 to 14, and who survived was 11 months; IQR 8 to 17 (Wilcoxon rank-sum test, P<0.001).

Figure S4. Cumulative hazard of death, according to age group

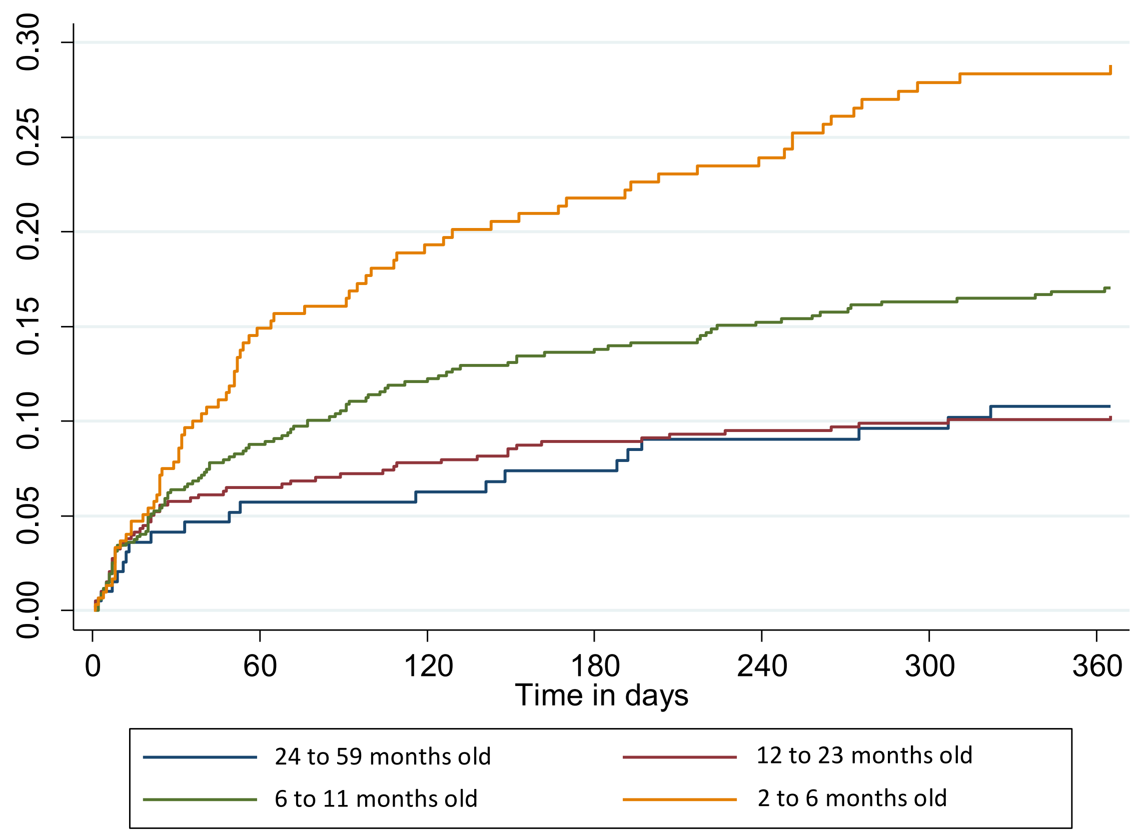


```
Cox regression -- Breslow method for ties
No. of subjects =          1778                  Number of obs   =       1778
No. of failures =           257
Time at risk    =      557599.5 days
                                                 LR chi2(3)      =      36.53
Log likelihood  =    -1880.3855                  Prob > chi2     =     0.0000
------------------------------------------------------------------------------
          _t | Haz. Ratio   Std. Err.      z    P>|z|     [95% Conf. Interval]
-------------+----------------------------------------------------------------
 24 to 59 m  |    Ref
 12 to 23 m  |   .9684414   .2516909    -0.12   0.902     .5819052    1.611738
  6 to 11 m  |    1.58426   .3865335     1.89   0.059     .9820808    2.555675
   2 to 5 m  |   2.631193   .6622552     3.84   0.000      1.60661    4.309183
------------------------------------------------------------------------------
```

REFERENCES FOR SUPPLEMENTARY APPENDIX

1. Scott JA, Bauni E, Moisi JC, et al. Profile: The Kilifi Health and Demographic Surveillance System (KHDSS). International journal of epidemiology 2012;41:650-7.
2. World Health Organization. Pocket book for hospital care of children: guidelines for the management of common illness with limited resources. 2005.
3. Guidelines of co-trimoxazole prophylaxis for HIV-related infections among children, adolescents and adults. 2006. (Accessed 1st August 2014, 2014, at http://www.who.int/hiv/pub/guidelines/ctxguidelines.pdf.)
4. Berkley JA, Lowe BS, Mwangi I, et al. Bacteraemia among children admitted to a rural hospital in Kenya. The New England journal of medicine 2005;352:39-47.
5. Moisi JC, Gatakaa H, Berkley JA, et al. Excess child mortality after discharge from hospital in Kilifi, Kenya: a retrospective cohort analysis. Bulletin of the World Health Organization 2011;89:725-32, 32A.

TRIAL PROTOCOL

Randomised, double blind, placebo-controlled trial of co-trimoxazole prophylaxis among HIV-uninfected children with severe malnutrition
(CTX Study)

Sponsor: University of Oxford

Principal Investigator(s): Dr. James Berkley

Local Safety Monitor: Dr Mary Mwangome

Version Number and Date: Version 3.1 6th March 2011

	**ID/Registration No.**
**KEMRI Scientific Steering Committee**	SSC 1562
**Kenya National Ethical Review Committee**	SSC 1562
**Oxford University Tropical Ethical Review Committee**	18-09
**ClinicalTrials.gov**	NCT00934492
**Kenya Pharmacy & Poisons Board Registration**	ECCT/09/06/01
**National Quality Control Laboratory Product Approval**	CAN/2009/491; CAN/2009/492

Table of Contents. **Page**

1. Lay Summary	6
2. Protocol Summary	7
3. Key Roles	8
4. Background and Scientific Rationale	9
5. Study Design	13
6. Study Population	13
7. Sensitisation and Recruitment Procedures	13
8. Community Engagement Strategy	14
9. Study Enrolment And Schedule Of Follow-Up Visits	14
10. Investigational Product	16
11. Assessment of Scientific Objectives	17
12. Assessment of Safety	18
13. Adverse Event Monitoring	19
14. Clinical Monitoring Structure	20
15. Statistical Considerations	21
16. Data Management	23
17. Quality Control and Quality Assurance	24
18. Ethics and Protection of Human Subjects	24
19. Informed Consent Process	24
20. Subject Confidentiality	25
21. Data Handling and Record Keeping	26
22. Protocol Deviations	26
23. Plans For Distribution Of Research Findings	26
24. References	26
25. Informed Consent Information and Forms	32

Statement of Compliance

This trial will be conducted in compliance with the protocol, International Conference on Harmonisation Good Clinical Practice E6 (ICH-GCP) and the EU Clinical Trials Directive (2001/20/EEC).

SIGNATURE PAGE

The signature below constitutes the approval of this protocol and the attachments, and provides the necessary assurances that this trial will be conducted according to all stipulations of the protocol, including all statements regarding confidentiality, and according to local legal and regulatory requirements and ICH guidelines.

Principal Investigator:

Signed: ____________________________________ Date: ________________

List of Abbreviations

AE	Adverse event
CGMR-C	Centre for Geographic Medicine Research- Coast
CRF	Case Report Form
CRP	'C'-Reactive Protein
CTF	Clinical Trials Facility
DSMC	Data Safety Monitoring Committee
DSS	Demographic Surveillance System
EU	European Union
GCP	Good Clinical Practice
ICH	International Conference on Harmonization
KEMRI	Kenya Medical Research Institute
LPS	Lipopolysaccharide (endotoxin)
LSM	Local Safety Monitor
MUAC	Mid-Upper Arm Circumference
OXTREC	Oxford Tropical Research Ethics Committee
PI	Principal Investigator
SAE	Serious Adverse Event
SOPs	Standard Operating Procedures
SUSAR	Suspected Unexpected Serious Adverse Reaction
TNF	Tumour Necrosis Factor
WHZ	Weight for Height Z-score

1. Lay Summary

What is the problem?

Malnutrition is one of the most important risk factors for childhood death in developing countries. Malnourished children are at greatly increased risk of death from infectious diseases in the community, in hospital, and after discharge from hospital. This is because malnutrition makes surface defences such as the skin and lining of the lungs and intestines weak, and causes the immune system to malfunction. In other conditions where the immune system is not working properly, such as HIV, long-term, low-dose daily antibiotics are highly effective in reducing mortality. The most commonly used is an antibiotic called cotrimoxazole (Septrin). It is not known if this might be effective in malnourished children.

What question are we trying to answer?

We would like to find out whether daily cotrimoxazole prophylaxis can reduce mortality among severely malnourished children who are not infected with HIV.

Where is the study taking place?

The study will take place in Kenya, at Coast Provincial General Hospital, Mombasa, Malindi District Hospital and Kilifi District Hospital.

How many people does it involve and how are they selected?

The study aims will involve **1850** children with malnutrition at three hospitals in Kenya. Eligible patients with severe malnutrition will be approached for consent after the initial stabilization phase using the standard clinical care for management of severe malnutrition. The patients will then be randomly allocated to receive cotrimoxazole or placebo. Neither the patient nor the study staff will have a way of knowing whether the patient is receiving the active drug or the placebo, until the study ends. Both look similar in colour, size and packaging.

What does the study involve for those who are in it?

Children who participate in the study will be given cotrimoxazole or an identical placebo for six months. The medicine will be taken once a day and will be given in monthly supplies. We will measure the child's weight and height at each visit and obtain samples of blood, stool, and a nasal swab at admission, and after three and six months. We will also follow the child up to one year.

What are the benefits and risks/ costs of the study for those involved?

There are no financial benefits to the participants for involvement in the study. Children will benefit from close observation and free medical care for common ailments according to current Kenyan national guidelines.

How will the study benefit society?

This study will help improve the quality of life and survival of children with malnutrition.

When does the study start and finish?

Q4 2009 to Q4 2014

2. Protocol Summary

Malnutrition is the most important overall risk factor for childhood death in developing countries. Severely malnourished children are at greatly increased risk of death from infectious diseases in the community, in hospital and following discharge. Among children treated for severe malnutrition in Africa, mortality following discharge from hospitals ranges between 8% and 41%. Cotrimoxazole is a licensed, cheap and widely available antibiotic with an established safety profile used for prophylaxis in African populations. Cotrimoxazole prophylaxis dramatically reduces mortality among children with HIV, irrespective of the degree of immune suppression. The primary effect appears to be in reducing bacterial infection, especially pneumonia. It is widely used in developed countries among children with other immune deficiencies to prevent infection. Children with severe malnutrition are also immune deficient, as evidenced by their susceptibility to infectious diseases, and may therefore benefit from daily antimicrobial prophylaxis.

Population: Children with severe malnutrition who are not HIV-infected.
Number of Sites: 4
Study Duration: 5 years
Description of Intervention: A daily dose of either cotrimoxazole or placebo for six months
Objectives:

Primary:

To determine the efficacy of daily prophylaxis with cotrimoxazole in reducing mortality among HIV-uninfected, severely malnourished children following initiation of nutritional rehabilitation.

Secondary:

To determine the toxicity associated with cotrimoxazole prophylaxis

To determine effects of cotrimoxazole prophylaxis on frequency of readmission to hospital

To determine effects of cotrimoxazole prophylaxis on causes of readmission and death

To determine effects of cotrimoxazole prophylaxis on growth

To determine the effects of cotrimoxazole on microbial populations and antibiotic or antimalarial resistance

Description of Study Design: Randomised, Placebo-Controlled and Double Blinded Trial

3. KEY ROLES

Principal Investigator

Dr. James Berkley (consultant paediatrician in infectious diseases and immunology, PI) [1,2]
jberkley@kilifi.kemri-wellcome.org

Co-Investigators

Johnstone Thitiri (study coordinator) [1] jthitiri@kilifi.kemri-wellcome.org

Moses Ngari (data manager) [1] mngari@kilifi.kemri-wellcome.org

Judd Walson (infectious diseases and HIV specialist, clinical trialist) [3,4] walson@u.washington.edu

Twahir Hemed (consultant paediatrician, Mombasa) [5] amihst@yahoo.co.uk

Victor Bandika (consultant paediatrician, Mombasa) [5] bandikav@yahoo.com

Morris Buni (District Medical Officer of Health, Malindi) [6] morrisbuni@yahoo.com

Dr Florence Kapanga (consultant paediatrician, Malindi) [6]

Ms Laura Mwakela (clinical officer) [1] lmwakela@kilifi.kemri-wellcome.org
Mrs Fauzat Hamid (clinical officer) [1] fhamid@kilifi.kemri-wellcome.org
Ms Rehema Ali (research nurse) [1] rali@kilifi.kemri-wellcome.org
Kathryn Maitland (consultant paediatrician, clinical trialist) [1,7] kmaitland@kilifi.kemri-wellcome.org
Roma Chilengi (head of clinical trials KEMRI Kilifi) [1,2] rchilengi@kilifi.kemri-wellcome.org
Greg Fegan [1,2] (trial statistician) gfegan@kilifi.kemri-wellcome.org
Institutions:

1. KEMRI/Wellcome Research Programme, CGMR-Coast, PO Box 230, Kilifi, 80108, Kenya.
2. Centre for Clinical Vaccinology and Tropical Medicine, University of Oxford, Oxford, UK
3. KEMRI CRC, Nairobi, Kenya.
4. University of Washington, Seattle, USA
5. Coast Provincial General Hospital, Mombasa, Kenya
6. Malindi District Hospital, Malindi, Kenya
7. Imperial College, London, UK

Sponsor: University of Oxford
TSC Chair: Prof. Diana Gibb
DSMC Chair: Prof. Tim Peto
Monitor: The Kilifi Clinical Trials Facility monitoring team
Study sites: Kilifi District Hospital, Malindi District Hospital and Coast Provincial General Hospital, Mombasa.
Clinical laboratories: KEMRI/Wellcome Trust Research Programme, Kilifi, Kenya
Local safety monitor: Dr Mary Mwangome, Kilifi, Kenya

4. BACKGROUND INFORMATION AND SCIENTIFIC RATIONALE

Malnutrition is the most important risk factor for childhood death in developing countries.[1, 2] Severely malnourished children are at greatly increased risk of death from infectious diseases in the community,[3-5] in hospital[6-8] and following discharge.[8-12] Cohort studies in Bangladesh, Malawi and Uganda suggest an annual mortality of more than 100 per 1000 at mid-upper arm circumferences (MUAC) less than 11cm.[3, 13-15] Risks probably vary between diseases: weight for age is associated with different relative risks of death from diarrhoea, pneumonia, malaria and measles.[16] Malnutrition and infection are synergistic, in part because malnutrition causes secondary immune deficiency,[8, 17-19] whilst infections cause losses and diversion of nutrients.[3, 17, 20, 21] The 'vicious cycle' between malnutrition and infection is exacerbated by a high level of exposure to pathogens in resource-poor communities.

Inpatient mortality

Admission to hospital with severe malnutrition commonly has a high case fatality,[22-24] even after exclusion of HIV.[25] This may be improved to some extent by adherence to WHO recommended management,[22, 23] but the direct application of these guidelines to all contexts is controversial. [26] Children are usually admitted to hospital because they are severely ill rather than for malnutrition alone. Children may die early during admission because of late presentation, infection, shock or metabolic disturbances.[24] However, many deaths occur later during admission and the associated factors are less clear but may include failure of initial antimicrobial therapy and nosocomial infection.[24, 27-30]

Post-discharge mortality

Nutritional status is a principal determinant of mortality following discharge from hospital. Among Bangladeshi children with diarrhoea, severe wasting at admission was associated with a post discharge mortality of 25% compared to 5.5% in better nourished children.[31] In the Gambia, even moderate underweight was associated with a rate ratio of 3.2 for death.[32] Among children treated for severe malnutrition, mortality following discharge from hospitals ranges between 8% and 41%.[10, 33-35] Deaths in these studies typically occur within 6 months.[10, 11, 31, 33] Lower mortality rates are seen among uncomplicated cases following treatment outside hospital-based facilities,[9, 36] and were seen in studies in the Caribbean,[8, 37] which may reflect the importance of exposure to infection.[18, 38]

The factors associated with post-discharge mortality are likely to include incomplete correction of nutritional deficiencies, a diet inadequate for immunological recovery, high burden of exposure to pathogens, recurrent infection due to poor immune status and abject poverty.[36, 39] Carers may have

considerable pressures for early discharge: loss of income, catastrophic costs of healthcare [40] and competing demands to care for siblings.[36]

Effects of infection on nutritional status

Clinical and sub-clinical infections have multiple effects on nutrient intake, absorption, secretion, diversion, catabolism and expenditure.[20] Diarrhoea is associated with malabsorption and marked losses of protein, vitamin A, zinc and other micronutrients.[41, 42] All infections are associated with net protein loss with diversion of amino acids to acute phase and immune response proteins.[43-46] Activation of inflammatory cascades also causes reduced appetite and loss of lean tissue and fat.[20, 38, 47] Fever is associated with an increased resting energy expenditure of 7 to 13% per degree Centrigrade.[48-50] Severely malnourished children may expend less energy and protein because of reductive adaptation,[44] but nutritional costs of infection may impair catch-up growth.[32, 38] Prevention of clinical and sub-clinical infection may therefore lessen immune activation and consequently reduce nutrient loss and wastage.

Effects of malnutrition on immunity

A wide variety of immunological abnormalities are reported among children with malnutrition. The strongest evidence of immune deficiency is increased susceptibility, severity and duration of infection,[8, 16, 51-58] There is reduced gut and respiratory mucosal integrity resulting in increased permeability, mucosal immune abnormalities[59-61] and reduced gastric acidity.[62, 63] The most consistently reported systemic immune abnormalities (in the absence of HIV infection) include thymic and lymphoid atrophy,[58] reduced T cell numbers,[64-67] impaired *in vivo* and *in vitro* tests of T cell activation and regulation.[7, 67-70] Phagocytic and bactericidal functions, and complement systems are also impaired.[71-74]

Following therapeutic feeding, complete recovery of barrier and measured immune function lags behind anthropometric improvement.[61, 64, 75-78] There is also evidence of persistent immune stimulation not corrected by short term antibiotics or nutritional therapy,[79] possibly related to continued exposure pathogens.[38] The main hypothesis underlying this project is that severely malnourished children remain vulnerable to life-threatening infection because of ongoing immune and barrier dysfunction, and a high exposure to pathogens.

Cotrimoxazole

Cotrimoxazole is a synthetic antibacterial combination that blocks two steps of folate metabolism involved in the biosynthesis of nucleic acids and proteins essential to many bacteria and some parasites, including Plasmodium falciparum. It is cheap, widely available and has an established safety profile in African populations. Among 541 HIV-infected Zambian children, cotrimoxazole prophylaxis dramatically reduced mortality (HR 0.57).[80, 81] Similar effects are reported among HIV-infected adults in Uganda[82] and South Africa.[83] In Cote d'Ivoire, cotrimoxazole prophylaxis was associated with sustained improved in anthropometric status by 3 months.[84, 85]

Apart from HIV, a 5 day course of cotrimoxazole treatment is currently recommended by WHO for children with severe malnutrition without complications.[86] Among children with measles in Guinea Bissau, cotrimoxazole prophylaxis almost eliminated pneumonia (OR 0.08) and was associated with greater weight gain.[87] Cotrimoxazole prophylaxis is used in developed countries for children with

primary and secondary immunodeficiency, especially among children with defective phagocytic or neutrophil function such as chronic granulomatous disease (CGD).[88-91]

The effect of cotrimoxazole on mortality appears to be mainly from prevention of bacterial infections such as pneumonia,[81, 92] often despite antimicrobial resistance.[80, 82] There is mixed evidence regarding an effect on diarrhoea: there were non-significant effects in the Zambian HIV study[80] and the Guinea-Bissau measles study;[87] there was a significant reduction in diarrhoea among Ugandan HIV-infected adults and their children.[82, 93] Malaria was effectively prevented by cotrimoxazole among HIV-uninfected children in Mali (99.5% protective efficacy)[94] and among HIV-infected adults and their children in Uganda (IRR 0.28).[82]

The protective efficacy of cotrimoxazole against death in HIV is striking given that it does not target the underlying immunodeficiency. There may be another immunological effect. For example, it has been argued that cotrimoxazole is immuno-modulatory and may be beneficial in rheumatoid arthritis.[95] Although, cotrimoxazole shares some properties of anti-folate anti-inflammatory drugs, this is unlikely to account for the effect seen. High levels of sulphamethoxazole and trimethoprim are found within granulocytes[96, 97] and there is evidence that cotrimoxazole specifically enhances intracellular bacterial killing.[98, 99] **This project will investigate the effect of cotrimoxazole prophylaxis on granulocyte (neutrophil) activation, phagocytosis and bacterial killing.**

Toxicity

Cotrimoxazole is associated with potentially important adverse effects including rash, neutropaenia and liver toxicity. In the Zambian HIV trial, grade 3 or 4 toxicity occurred in 6% (mostly neutropenia). The frequencies of events were similar among the active and placebo groups.[80] A meta-analysis in adults (n=1,476) suggests a small, non-significant increase in adverse events for cotrimoxazole versus placebo.[100] All resolved on withdrawal. In 9 studies among adults and children in Africa, significant adverse events occurred in 0.3 to 6%.[101]

Microbial ecology and antimicrobial resistance

There are limited data on effects of cotrimoxazole prophylaxis on bacterial ecology. In Cape Town, nasal carriage of *S. aureus* was reported to be increased in children taking cotrimoxazole.[102] However, recently at the same site, amongst 203 HIV-infected children, only minor differences were found in organisms isolated from nasal swabs between those receiving cotrimoxazole and those not: cotrimoxazole was associated with less *Pseudomonas aeruginosa* and Acinetobacter sp., whilst carriage of common respiratory pathogens (*S. pneumoniae, H. influenzae, M. catarrhalis* and *S.aureus)* were unaffected. In KwaZulu-Natal, no association was found between cotrimoxazole and carriage of *S. aureus or S. pneumoniae.* American data on cotrimoxazole and pneumococcal carriage in children are also inconsistent.[103, 104]

There is concern that widespread use of cotrimoxazole may exacerbate resistance. Selection for penicillin non-susceptibility in pneumococci is an important potential concern.[104-106] In Cape Town, cotrimoxazole prophylaxis was associated with a non-significant increase in nasal carriage of pathogens resistant to cotrimoxazole (91 vs. 86%, P=0.065) and specifically of methicillin resistant *S. aureus*. Pneumococcal resistance was associated with a lack of history of hospitalisation, but not cotrimoxazole. Increased carriage of resistant enteric bacteria has been reported among adults[82, 93, 107-109] but there are no data from African children.

There is potential for cross-resistance with malaria.[110, 111] Cotrimoxazole treatment courses are associated with selection of resistance-related polymorphisms.[112] However, among HIV-uninfected

children in Mali, there was no increase in anti-folate resistant mutations after at least one month of cotrimoxazole prophylaxis.[94] In Uganda, cotrimoxazole prophylaxis was associated with fewer episodes of infection with DHFR/DHPS quintuple mutants among HIV-infected adults and their household contacts.[82, 93, 113] Since anti-folate antimalarials may be used in combination with artemisinin derivatives or for intermittent presumptive treatment, further investigation is needed.

Economics

Cotrimoxazole prophylaxis has been economically evaluated only in the context of HIV. Among Zambian children in the CHAP trial, cotrimoxazole prophylaxis was associated with incremental cost-effectiveness ratios of US$72 per life-year saved and US$53 per DALY averted.[114] Among adults in Ivory Coast an incremental cost per year of life gained of US$ 240 is reported.[115] Data from Uganda suggest cotrimoxazole is initiated early, before HIV disease progression.[116]

OBJECTIVES

Primary:

To determine the efficacy of daily prophylaxis with cotrimoxazole in reducing mortality among HIV-uninfected, severely malnourished children following initiation of nutritional rehabilitation.

Secondary:

1. To determine the toxicity associated with cotrimoxazole prophylaxis
2. To determine the effects of cotrimoxazole prophylaxis on rates of hospital readmission
3. To determine the effects of cotrimoxazole prophylaxis on causes of hospital readmission and death
4. To determine effects of cotrimoxazole prophylaxis on growth (weight, height, MUAC).
5. To determine the effects of cotrimoxazole prophylaxis on microbial populations and antibiotic or antimalarial resistance
6. To determine the effects of cotrimoxazole prophylaxis on inflammation, immune activation, **neutrophil function** and gut bacterial translocation
7. To collect data that allows an economic evaluation of cotrimoxazole prophylaxis

ENDPOINTS:

Primary: Death

Secondary:

- Toxicity: grade 3 or 4 clinical or laboratory adverse events causally related to the trial drug.
- Frequency of hospital re-admission
- Causes of hospital readmission and death
- Growth
- Antimicrobial species and sensitivities of bacteria and malaria parasites
- Marker of inflammation, immune activation and **function**

5. STUDY DESIGN

Randomised, double blind, placebo-controlled trial with 90% power to detect a hazard ratio of 0.66 at the 5% significance level from baseline mortality at six months of **15%**. Children will be recruited as inpatients after initial stabilisation treatment and receive the investigational product for six months. Follow-up will be for 1 year or trial closure. The primary endpoint is death. Secondary endpoints are hospital re-admission, growth and antimicrobial sensitivities.

Study sites: Kilifi District Hospital, Coast Provincial General Hospital, **Mbagathi District Hospital, Nairobi,** Malindi District Hospital.

Sample size: 1850 patients randomised into active and control groups.

Randomisation: Masked trial drugs will be labelled with sequential study numbers according to a prepared blocked randomisation list before the trial begins. Randomisation codes linking allocation to study number will be held by the trial statistician and chair of the DSMC. Children will be allocated study numbers sequentially within each site, thus randomly allocating the trial drug and maintaining blinding.

6. STUDY POPULATION

Inclusion Criteria:

- Age 2 months to 5 years
- Admitted to hospital and completed the stabilisation phase of treatment (defined in current WHO guidelines, see below).[86]
- Severe malnutrition: age 6 months to 5 years: **MUAC <11.5cm**; age 2 to 6 months: **MUAC <11cm**; or kwashiorkor at any age (defined in current WHO guidelines) .[86]
- HIV rapid test negative, or if under 18 months, PCR negative and no longer breastfeeding for at least 6 weeks
- Planning to remain within study area and willing to come for all protocol specified visits.

Exclusion Criteria:

- Refusal to give informed consent
- Cotrimoxazole is specifically contra-indicated (e.g. porphyria)
- Known hypersensitivity reaction to sulpha drugs or trimethoprim

7. SENSITISATION AND RECRUITMENT PROCEDURES

Pre-trial sensitisation

Nursing, nutritional and medical staff at the three sites will be informed about the trial and training in management of malnutrition will be undertaken. Parents and carers of children will be informed about the trial during the initial period of treatment and stabilisation after admission and before recruitment.

Recruitment

1850 children will be recruited during 3 years in Mombasa, Malindi and Kilifi. Recruitment will be after the initial stabilisation phase (correction of hypothermia, hypoglycaemia, shock, dehydration, provision of broad spectrum antibiotics, initial nutritional supplementation and recovery of appetite; usually between days 3 and 7, as defined in the current WHO guidelines: resolution of hypothermia, hypoglycaemia, dehydration and shock AND the child has regained appetite. If the child has kwashiorkor, the oedema must have started to resolve.[86] Screening and information-giving will take place during this initial period. The study will be explained to parents and carers following admission, during the stabilisation period, creating a staged informed consent process.

8. COMMUNITY ENGAGEMENT STRATEGY

Community engagement will be through the inpatient wards and follow up clinics at the hospital trial sites; through the District and Provincial Medical Officers of Health offices – through these means, referral clinics and hospitals will be informed; and in Kilifi, through the existing KEMRI-Community Representatives (KCRs).

9. STUDY ENROLLMENT AND SCHEDULE OF FOLLOW-UP VISITS

Screening

Screening will take place within the four hospitals. All children admitted to hospital with non surgical conditions will be considered. The screening process will be based on the severe malnutrition criteria which is part of any hospital admission routine. HIV testing by rapid antibody test will be offered at admission to all potentially eligible admissions. PCR will be performed for children with positive antibody tests among infants less than one year old (dried blood spot on filter paper will be collected at the same time as the blood for rapid test). Through this, potential participants i.e. those with severe malnutrition will be identified, brought to the attention of the study team and given information about the study. Once stabilised, parents and carers will be invited to enrol**.**

Study specific procedures will be communicated only to the parents/guardians of potential participants in order to obtain informed consent. Information on benefits and risks will be provided to parents or carers in their own language and written informed consent sought. A screening log book will be maintained to capture all participants considered for the study and reasons for non-inclusion.

Enrolment

Upon satisfaction of the inclusion criteria and completion of informed consent forms, study ID number will be allocated. Baseline data of prognostic importance will be collected, including vaccination status. Children will be managed to the best standard available in each facility, in line with WHO recommendations. The decision to discharge made by the clinical team normally working on the unit. Children will be assessed by MUAC and the presence of nutritional oedema recorded. Nasal swabs, faecal samples and blood for haemoglobin, malaria slide, a dried blood spot, and immunological markers (e.g. CRP, TNF-α, beta-2-microglobulin, anti-LPS antibody levels) will be collected (5ml). Carers will be issued with a study card containing their study number and appropriate advice. On discharge, families will be given their supply of study drug and provided clear instructions on how to take it. Food supplements and/or nutritional counselling that are the current

standard of care at each institution will also be given at discharge. The family will be accompanied home by a study fieldworker who will record the exact household location by GPS.

Follow up

Follow up will be monthly for six months and then two-monthly to one year. Carers will be provided with fares to attend follow up for anthropometry, pill counting and compliance counselling. The study drug will be dispensed monthly for the first six months and carers asked to return the empty blister packets. A health and medication questionnaire will be administered detailing outpatient and dispensary visits, and medication not prescribed by the study team. Children judged to have significant illness by the assessing nurse will be referred to the outpatient clinic or hospital nutritionist. Children who default a follow up visit will be telephoned if a mobile number is available and visited at home by study fieldworkers. Nasal swabs, faecal samples and blood for full blood count, malaria slide, a dried blood spot, immunological markers (e.g. CRP, TNF-α, beta-2-microglobulin, LPS) and **tests of neutrophil function** will be collected at 2, 6 and 12 months. Plasma and cells will be stored at -80°C to allow further investigations to be conducted. Approval for any further investigations on these stored samples will require approval by KEMRI SSC and ERC.

Summary of study events

ACTIVITY	SCREENING	RECRUITMENT	MONTH 1	MONTH 2	MONTH 3	MONTH 4	MONTH 5	MONTH 6	2-MONTHLY FOLLOW UP TO MONTH 12	UN-SCHEDULED VISITS / RE-ADMISSION
Child admitted to hospital	X									
Screening for severe malnutrition	X									
Standard case management	X	X								X
Eligible children Identified	X									
Provide information about the study	X									
Anthropometric measurements	X	X	X	X	X	X	X	X	X	X
Informed Consent		X								
Eligible children randomised		X								
Baseline data collection		X								
Counselling on compliance etc			X	X	X	X	X	X		
Supply of study drug to last one month		X	X	X	X	X	X			
Collection of Lab samples*		X		X				X	X at 12 months	X
Monitoring of Adverse Events		X	X	X	X	X	X	X	X	X

* Lab samples at month 0, 2, 6 and 12 months include nasal swabs, faecal samples and blood for haemoglobin, and immunological markers (5ml).

Carers will be asked to come to the hospital should the child be unwell. Field workers and study staff will be trained on identification of admitted participants and reporting of adverse events required by this protocol. Study participants who are readmitted to hospital will undergo standardised clinical assessment including history, examination, full blood count, malaria slide, dried blood spot for malaria genotyping, blood culture, urine culture (if dipstick positive for leucocytes or nitrite) and stool culture (if diarrhoea). Other investigations such as liver or renal function testing will be performed as clinically indicated. First line antimalarial treatment will be artemether-lumefantrine (non-severe) or quinine (severe) as per national guidelines. Antibiotic treatment will also follow WHO and Kenyan guidelines.

10. INVESTIGATIONAL PRODUCT

Cotrimoxazole

Cotrimoxazole dispersible tablets (240mg) and identical placebos will be manufactured to GMP standards by Cosmos Pharmaceuticals Ltd, Nairobi. Masked trial drug will be block randomised with sequential study numbers. Sealed randomisation codes will be held by the trial statistician and DSMC chair. Dosing will be as per Kenyan recommendations for prophylaxis in HIV: age 2 to 6 months: trimethoprim 20mg/sulphamethoxazole 100 mg and age 6 months to 5 years: trimethoprim 40 mg/sulphamethoxazole 200mg. [101] The dispersible tablets may be taken with water or mixed with feeds.

Placebo control tablets

The placebo will be dispersible tablets and identical to the cotrimoxazole, is manufactured to GMP standards by Cosmos Pharmaceuticals Ltd, Nairobi. The dosing schedule will be exactly the same as for the study drug but the active ingredients replaced by starch or similar inert substance. The dispersible tablets may be taken with water or mixed with feeds.

11. ASSESSMENT OF SCIENTIFIC OBJECTIVES

The following are key evaluation criteria and information on these will systematically be collected on Case Record Forms (CRF).

Primary Evaluation Criteria

- <u>Death</u>: Enhanced surveillance will be implemented on the study cohort to ensure that any death occurring is reported. A separate verbal autopsy form will be completed for deaths outside hospital later on following a traditionally acceptable period of time, and these will be used to evaluate the causes of death.

Secondary Evaluation Criteria

- <u>Toxicity:</u> Our safety surveillance system will be set to detect and document adverse events. Grades 3 and 4 clinical and laboratory abnormalities will be reported (see table below).
- <u>Hospital admission</u>: Rates of hospital admissions will be compared between the two groups.
- <u>Causes of readmissions and deaths</u>: including aetiology where possible, major clinical

syndromes (pneumonia, gastroenteritis, sepsis etc) and proven bacterial infection and malaria requiring admission will be determined.

- Growth: All study children will be followed up to evaluate changes in weight, MUAC and height at baseline, 2, 6, and 12 months.
- Microbial ecology and resistance: Comparison of species and their antimicrobial resistance for invasive isolates, and nasal and stool carriage will be obtained at baseline, 2, 6 and 12 months. The prevalence of DHRF and DHPS mutations from dried blood spots, among the two groups when malaria parasites are detected and antimicrobial resistance profiles of carriage and invasive bacteria will be reported.
- Economic evaluation: Data on outpatient visits, hospital readmissions and deaths will be collected to allow an economic analysis in terms of cost per life-year saved and disability adjusted life years (DALYs).

Exploratory Evaluation Criteria

The following aspects will also be evaluated in an exploratory manner to determine the effects of cotrimoxazole prophylaxis on immune activation **and neutrophil function** and gut translocation:

- Inflammatory and immunological effects: The profile of inflammatory and immune activation markers, including C-reactive protein, TNF-α, inflammatory cytokines, beta-2-microglobulin, LPS and anti-LPS antibodies (markers of gut integrity) will be compared between the two groups at baseline and 6 months. **Neutrophil activation, phagocytosis, oxidative burst and microbial killing will be assessed by multi-parameter flow cytometry and assays of fluorescently-labelled E. coli particle uptake; oxidation of fluorescent substrate following *in vitro* LPS challenge and the neutrophil-dependent decrease in viable E. coli count.** Plasma and cells will be stored for future analyses subject to SSC and ERC approval.

12. ASSESSMENT OF SAFETY

All adverse events (AE) occurring in participants during the trial will be documented and reported as described below.

Safety reporting definitions

Adverse Event (AE)

An AE is any untoward medical occurrence that may occur during or after administration of the investigational product. The AE may or may not have a causal relationship with the product. The definition includes intercurrent illnesses, injuries, exacerbation of pre-existing conditions, and events occurring as a result of product misuse or overdose.

Toxicity

Grade 3 and 4 clinical and laboratory toxicities are pre-specified for allergy, rash, anaemia, neutropenia and liver dysfunction as these are what were seen in previous studies (see table below). A change in a laboratory variable alone will be reported as an AE if it is classified as grade 3 or 4 toxicity. A clinical toxicity may be an AE or an SAE depending on severity.

Toxicity	***Grade 3***	***Grade 4***
Allergy*	Mild to moderate urticaria	Severe urticaria,<br>Angiooedema, Anaphylaxis<br>Bronchospasm requiring treatment
Cutaneous*	Vesiculation or ulceration…<br>(the skin surface is broken)	Exfoliative dermatitis,<br>(the skin is coming off)<br>Stevens- Johnson syndrome,<br>Toxic epidermal necrolysis
Anaemia**	Haemoglobin 4 to 5g/dl,<br>not transfused.	Haemoglobin <4 g/dl or<br>Haemoglobin <5 g/dl with evidence of shock, respiratory distress or cardiac failure.
Neutropenia*	Neutrophil count $<0.4 \times 10^9$ /L	Neutrophil count $<0.25 \times 10^9$ /L
Liver dysfunction*	Transaminases or gamma-glutamyl transferase (GGT)<br>>10x upper limit of normal:<br>ALT 370 to 555 IU/L<br>AST 420 to 630 IU/L<br>GGT 460 to 690 IU/L<br>Bilirubin 3.0 to7.5 x normal:<br>50 to 128 µmol/L	Transaminases or gamma-glutamyl transferase (GGT)<br>>15 x upper limit of normal:<br>ALT >555 IU/L<br>AST >630 IU/L<br>GGT >690 IU/L<br>Bilirubin >7.5 x normal:<br>Above 128 µmol/L

Sources:
* WHO CTX guidelines for HIV 2006: http://www.who.int/hiv/pub/guidelines/ctxguidelines.pdf
** WHO guidelines for management of sick children at the hospital level 2006.[86]

Serious Adverse Event (SAE)

An AE (whether or not considered related to the investigational product) resulting in any of the following outcomes is defined as an SAE:

- Death (from any cause at any time)
- Life-threatening event (i.e., the subject was, in the view of the investigator, at immediate risk of death from the event that occurred).
- Persistent or significant disability or incapacity (i.e., substantial disruption of ability to carry out normal life functions).
- Hospitalisation or an important medical event requiring medical or surgical intervention to prevent one of the outcomes listed above. Examples include severe adverse drug reactions such as severe allergic reaction or blood dyscrasias requiring intensive treatment in an emergency room or clinic.

Suspected and Unexpected Serious Adverse Reaction (SUSAR)

These are the reactions that are serious (as defined above) but not expected AND considered to be related to the investigational product. This definition captures the important events that are attributed to the product but do not follow known pattern of response to study product.

Adverse Events Assessment of Causality

An assessment of the relationship of the event to the study drug will be undertaken by the senior clinical investigators (JB and RC) who will remain blinded to study allocation. This interpretation will be based on the type of event, the relationship of the event to the time of administration, and the known biology of the intervention. The following are guidelines for assessing the relationship of administration of to the product to the AE:

Relationship	Is there a reasonable possibility that the AE may have been caused by the investigational product?
**No**	The AE is not causally related to administration of the study drug. There are other, more likely causes.
**Yes**	The AE is most likely to be causally related to the study drug.

SAE and SUSAR Reporting

All SAEs will be documented and reported by the site investigator to the study team within 7 days of the investigator becoming aware of the SAE. The investigator will compile a report of the SAE as a CRF which will include the name of reporting doctor and contact telephone number; study number; subject details (initials, sex, weight and age); nature of adverse event; date and time of event; other drug history; other relevant history; outcome and a judgement of causality.

For SAEs deemed by the investigator as causally related to the study product and all SUSARs, the investigators will formally report these to the LSM and DSMC within 7 days. For all other SAEs, a summary report of all SAEs will be sent the LSM, DSMC, the ethics committees (KEMRI and OXTREC) and sponsor (Oxford University) every 3 months.

Practical handling of adverse events

In the event of an abnormal clinical or laboratory finding by the study clinician, children will receive appropriate treatment according to Kenya national or WHO clinical guidelines, including admission for assessment and/or treatment where appropriate. Usual clinical practice for suspected reactions to cotrimoxazole will be followed. Where necessary for patient safety, this may include unblinding by the local safety monitor.

13. ADVERSE EVENT MONITORING

Data Safety and Monitoring Committee:

The role of the DSMC provides independent review and advice on the progress of the study. The focus of the DSMC is primarily safety of the study participants; the overall integrity of the study; its continued relevance and ability to answer the primary objective. Interim reviews of unblinded data will be conducted during the trial. The timing of the reviews will depend on recruitment and event rate and will therefore be at the discretion of the DSMC. The DSMC will operate mainly by teleconferencing at pre-specified regular intervals. The operations of the DSMC are separately detailed in a DSMC charter.

Halting Rules

A decision to discontinue recruitment, in all patients or in selected subgroups, may be made by the TSC on advice from the DSMC if the data provide proof beyond reasonable doubt that one of the treatment arms is better in terms of the primary outcome. The guiding statistical criterion for "proof beyond reasonable doubt" is the Haybittle-Peto criterion of a difference of at least 3 standard deviations in an interim analysis of a major endpoint. The TSC will then decide whether to amend or stop the trial before the end of the planned follow-up.

Role of the Local Safety Monitor

Dr. Mary Mwangome has been named as LSM on this study. She is an experienced and independent clinician who is locally accessible to the study investigators for consultation on management of some clinical cases. The LSM will also retain a sealed code break envelope and may rarely, at the advice of the DSMC or in extreme safety considerations, be able to un-blind specific individual allocation. This would however be clearly discussed and agreed beforehand with the DSMC.

14. CLINICAL MONITORING STRUCTURE

Site Monitoring Plan

Study monitoring will be conducted to ensure that the safety and conduct of the study complies with ICH Guidelines and the trial's standard operating procedures. The study will be monitored by the KEMRI/Wellcome Clinical Trials Facility Monitoring Team. The monitoring team comprises of trial coordinators and trial managers from the various trials currently running within the unit. To achieve independence during the monitoring, the monitors for this study will be trial coordinators/managers working on another trial. A comprehensive study initiation visit will be done at which all staff will be trained in protocol specific procedure, and check that all trial logistics are in place. Routine monitoring will be conducted every three months or more frequently if required. The monitor's role is part of the quality system that will ensure that entries relating to eligibility, randomisation and the primary outcome on the CRF are source verified, that the study file is maintained with updated master file documents, that all participants have dully completed informed consent and that staff on the study are following their SOP's in accordance to protocol and applicable regulatory norms. Reports from each study monitoring shall be submitted to the study investigators management team.

15. STATISTICAL CONSIDERATIONS

Determination of the Sample Size

Sample size is based on estimates of mortality using data from children age 2 months to 5 years resident in the Kilifi DSS area, admitted between 2002 and 2007. One year mortality among HIV-uninfected children meeting enrolment criteria is estimated at 15%, including children already enrolled into the trial at the stricter criteria of MUAC <11cm.

Power curves at 5% significance (two sided) for a baseline mortality of 15%, allowing for 5% loss to follow-up per year in each group, calculated using Analysis Resources for Trials for STATA (version

1.0.4, MRC Clinical Trials Unit) are shown below. A total sample of 1850 children recruited over 5 years at 3 sites is feasible and has 90% power to detect a hazard ratio of 0.66. For comparison, a hazard ratio of 0.57 was found in the Zambian CHAP study.

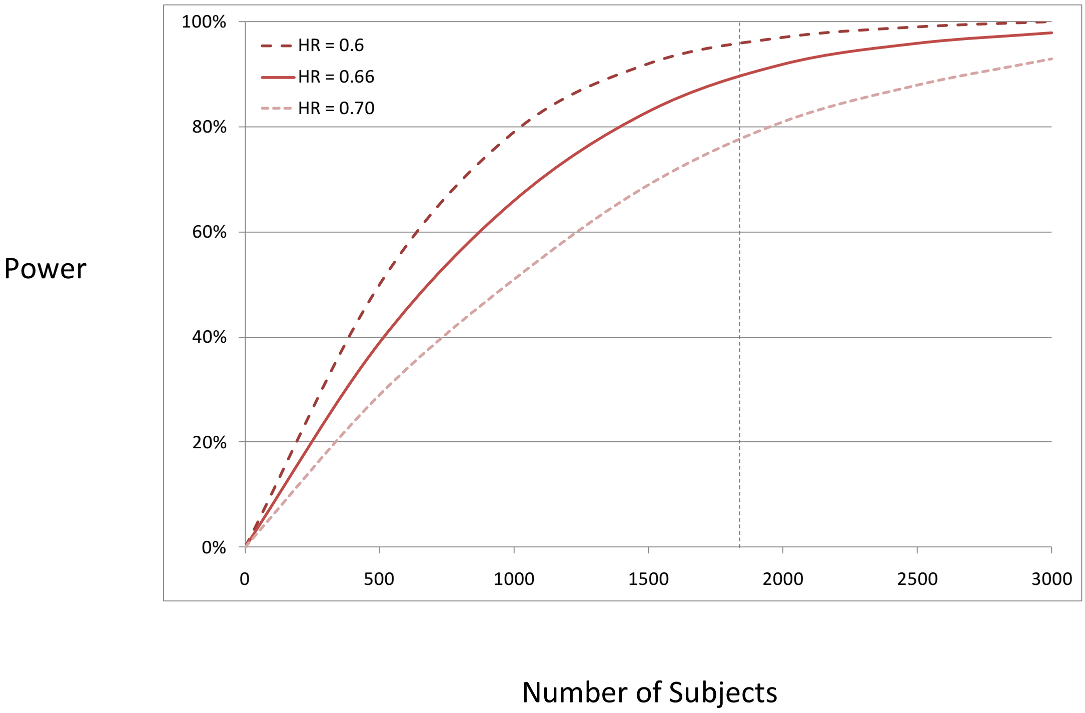

Expected analyses and outcomes

Analysis

A written analysis plan will be agreed by the study team the DSMC and TSC before unblinding. Time to event curves will be analysed using the Kaplan-Meier method. Hazard ratios for mortality at 6 and 12 months will be estimated using Cox proportional hazards regression and comparisons between randomised groups will be made using the log rank test. Heterogeneity across subgroups will be examined by interaction tests in Cox models. The incidence of toxicity and morbidity events will be estimated per child/year of follow up and compared using hazard ratios. Growth will be compared by time to established recovery targets using Kaplan-Meier survival analysis and by group comparisons at discharge, 6 and 12 months using a rank sum test and using appropriate statistical techniques to account for survivor bias. Proportions of bacterial isolates at each time point that are resistant to commonly used antibiotics will be compared using the chi square test. Where appropriate, statistical methods that account for multiple observations.

Microbial resistance and ecology

To investigate effects on microbial carriage and disease, stool and nasal samples and, where appropriate, readmission blood cultures will be inoculated on appropriate media to isolate staphylococci, streptococci (*including S. pneumoniae*), *H. influenzae, M. catarrhalis* and other Gram-negative organisms and tested for phenotypic resistance by standard methods. Malaria parasites from dried blood spots will be analysed for point mutations in codons 108, 51, 59, and 164 of DHFR and 436, 437, 540, 581, 613 of DHPS.[117, 118] Prevalence of carriage and resistance will be reported. All this work will be done in Kilifi

To examine effects on bacterial population diversity, DNA will be extracted from nasal and stool samples from a subset children receiving cotrimoxazole or placebo and transported to the Sanger Institute, UK. Universal bacterial primers that cover almost the complete 16S rRNA gene will be used. PCR products will be ligated into pGEM-T Easy Vectors (Promega) and transformed into high efficiency competent JM109 *E. coli* cells (Promega). These cells will then be grown on LB agar supplemented with the carbenicillin and subjected to blue-white screening. White colonies will be picked randomly, plasmid DNA purified and the 16S rRNA gene insert sequenced.

16. DATA MANAGEMENT

Source Documents

The routine hospital patient records shall be used according to existing systems at the hospitals. Additional source documents will be introduced in the form of bound books for screening logs, Informed consent forms, tracking forms, specimen transfer forms, product accountability logs etc. These will constitute the first place where information is written and the monitor shall check all CRF entries against the source documents. These will therefore be stored safely in and shall be made accessible to the study team and study monitor.

Case Record Forms:

Study specific data will be transcribed by the study nurses and coordinators to the paper case record forms. Each participant will thus have a CRF documents will all field necessary for evaluation of study outcomes to be taken to the ICT department for electronic. Before data entry, the coordinator and monitor shall check that all entries on CRF are backed by clear source data. Only individual duly authorized and documented as authorized signatories shall be allowed to make CRF entries.

Electronic Processing

Completed CRFs will be brought to the CTF data management department weekly where they will be logged in batches. All data will be double entered by two separate entry clerks working under a Data Manager will run regular reconciliation to derive the final study database. Access to the study database will be restricted and password protected.

The database will be developed in “OpenClinica” with features of GCP compliance. Specific access rights shall be tailor made to the function of the operators i.e. although the study investigators may view the data fields, they will not be able to make any entries or changes to the database. The system shall be able to generate automated queries and the data manager shall be able to generate some manual queries. All queries shall be passed through the study coordinator and clarified by the investigators and field staff with clear documentation.

The database shall maintain an audit trail system to be effected immediately the data lock time point is reached. The database lock time point will come after all participants have completed their scheduled follow up and all data queries have been answered.

17. QUALITY CONTROL AND QUALITY ASSURANCE

The investigators will draft a quality assurance system that will ensure that the trial is conducted to the highest standard and that the safety and well-being of study participants are protected and respected**.** We shall develop and use approved standard operating procedures for all key steps involved in the study starting with our approach to the study community, informed consent process, eligibility screening, enrolment, randomisation, clinical care, administration of study products, safety follow up and reporting as well as data processing.

All key staff, including field workers will be trained on their specific procedures before they can take any role on the study. The monitor and coordinator will check all CRF entries for completeness and accuracy; and these will further be checked by the senior investigators en route to data entry. Monitoring will constitute a key quality control activity for this study. Any systematic errors will require swift review and amendment of SOPs if necessary.

The DSMC will provide an independent oversight role looking into the progression of the study and making clear guiding recommendations to the TSC. The Local safety Monitor also provides another level of checks on the quality of clinical care provided to the participants.

18. ETHICS & PROTECTION OF HUMAN SUBJECTS

This protocol will be approved by the KEMRI/National Ethical Review Committee, Kenya and the Oxford Tropical Research Ethics Committee (OXTREC) before this study commences. This trial will be conducted in accordance with the current revision of the Declaration of Helsinki and the International Conference for Harmonisation Good Clinical Practice (ICH-GCP) regulations.

Our study does not introduce risks to participants beyond what they would normally face in their condition. The inconvenience and risks due to our study specific procedures are outweighed by the potential benefits to the individual participants as well as the community as a whole. Findings from this study may lead to recommendations that would significantly improve mortality and morbidity in malnourished children. Further, we shall work in close consultation with Social Behavioural Research group at KEMRI CGMRC that has required expertise when dealing with the study communities and they will guide the study team in that regard.

19. INFORMED CONSENT PROCESS

The study will be carried out in conformity to the ICH-GCP principles for informed consent. These principles will be stated and explained clearly in an informed consent SOP. This will be the basis for training staff involved in obtaining informed consent.

The parent or guardian will receive an explanation of the study by a member of the study team in private and in an appropriate language (see informed consent form) during the stabilisation period, creating a staged informed consent process. They will be given a chance to ask questions before written permission for their child to be included in the study is sought.

The quality assurance mechanisms of the Clinical Trial Facility will ensure that the procedures described in the Informed consent SOP are adhered to by checking during the monitoring process and ensuring that each study participant has dully completed consent form.

20. SUBJECT CONFIDENTIALITY

All records will be kept in a locked filing cabinet, which is accessed only by the investigators and the study nurse(s). All computer entry and networking programs will be done with coded numbers and initials only. Only the investigators, the clinical monitor, the ethics committees or any other regulatory agencies, at the request of the collaborator will have access to the records. Every effort will be taken to maintain confidentiality.

The study protocol, documentation, data and all other information generated will be held in strict confidence. Clinical information will not be released without written permission of the parents, except as necessary for monitoring. No information concerning the study or the data will be released to any unauthorized third party, without prior written approval of the sponsor.

21. DATA HANDLING AND RECORD KEEPING

A dedicated data manager will be employed with responsibility for receiving, entering, cleaning, querying, analysing and storing all data that accrues from the study. Patient data will be first collected in source documents which will be the origin of data recorded in a subject's CRFs created thereafter. The data in the CRFs will later be entered into an 'OpenClinica' database for storage pending analysis. Source documents will be retained for a minimum of 2 years from the end of the study in the institution's archive. CRFs will be retained for at least 10 years.

22. PROTOCOL DEVIATIONS

The investigator will conduct the trial in compliance with the protocol agreed to by the sponsor and which was given approval by the ethics committees. The investigator will sign the protocol to confirm agreement. The investigator will not implement any deviation from, or changes of the protocol without agreement by the sponsor and prior review and documented approval from ethics of an amendment, except where necessary to eliminate an immediate hazard(s) to trial subjects, or when the change(s) involve only logistical or administrative aspects of the trial (e.g., change in monitor(s), change of telephone number(s)).

The investigator, or person designated by the investigator, will document and explain any deviation from the approved protocol in the protocol deviation file. The investigator may implement a deviation from, or a change of, the protocol to eliminate an immediate hazard(s) to trial subjects without prior ethics approval. As soon as possible, the implemented deviation or change, the reasons for it, and, if appropriate, the proposed protocol amendment(s) should be submitted: to ethics for review and approval and, to the sponsor for agreement if required. A protocol deviations folder will contain documentation of all pre-planned deviations from the protocol and their justification. A protocol violations folder will contain documentation of unplanned protocol deviations.

23. PLANS FOR DISTRIBUTION OF RESEARCH FINDINGS TO STUDY COMMUNITY

Individual subjects will remain blinded to their study allocation at their last follow up visit. Results of the study will be fed back to the study community through the KEMRI community representatives, public meetings, district nutritionists of the districts involved and the follow up clinics in each site. Results will be shared nationally through presentation at the Kenya Paediatric Association annual scientific meeting.

24. References

1. Black RE, Morris SS, Bryce J. Where and why are 10 million children dying every year? Lancet. 2003 Jun 28;361(9376):2226-34.
2. Bryce J, Boschi-Pinto C, Shibuya K, Black RE. WHO estimates of the causes of death in children. Lancet. 2005 Mar 26-Apr 1;365(9465):1147-52.
3. Pelletier DL, Frongillo EA, Jr., Habicht JP. Epidemiologic evidence for a potentiating effect of malnutrition on child mortality. Am J Public Health. 1993 Aug;83(8):1130-3.
4. Schroeder DG, Brown KH. Nutritional status as a predictor of child survival: summarizing the association and quantifying its global impact. Bull World Health Organ. 1994;72(4):569-79.
5. Yoon PW, Black RE, Moulton LH, Becker S. The effect of malnutrition on the risk of diarrheal and respiratory mortality in children < 2 y of age in Cebu, Philippines. Am J Clin Nutr. 1997 Apr;65(4):1070-7.
6. Berkley J, Mwangi I, Griffiths K, Ahmed I, Mithwani S, English M, et al. Assessment of severe malnutrition among hospitalized children in rural Kenya: comparison of weight for height and mid upper arm circumference. Jama. 2005 Aug 3;294(5):591-7.
7. Allende LM, Corell A, Manzanares J, Madruga D, Marcos A, Madrono A, et al. Immunodeficiency associated with anorexia nervosa is secondary and improves after refeeding. Immunology. 1998 Aug;94(4):543-51.
8. Waterlow JC, Tomkins AM, Grantham-McGregor SM. Protein Energy Malnutrition. London: Edward Arnold; 1992.
9. Khanum S, Ashworth A, Huttly SR. Growth, morbidity, and mortality of children in Dhaka after treatment for severe malnutrition: a prospective study. Am J Clin Nutr. 1998 May;67(5):940-5.
10. Reneman L, Derwig J. Long-term prospects of malnourished children after rehabilitation at the Nutrition Rehabilitation Centre of St Mary's Hospital, Mumias, Kenya. J Trop Pediatr. 1997 Oct;43(5):293-6.
11. Roy SK, Chowdhury AK, Rahaman MM. Excess mortality among children discharged from hospital after treatment for diarrhoea in rural Bangladesh. Br Med J (Clin Res Ed). 1983 Oct 15;287(6399):1097-9.
12. West TE, Goetghebuer T, Milligan P, Mulholland EK, Weber MW. Long-term morbidity and mortality following hypoxaemic lower respiratory tract infection in Gambian children. Bull World Health Organ. 1999;77(2):144-8.
13. Alam N, Wojtyniak B, Rahaman MM. Anthropometric indicators and risk of death. Am J Clin Nutr. 1989 May;49(5):884-8.
14. Briend A, Wojtyniak B, Rowland MG. Arm circumference and other factors in children at high risk of death in rural Bangladesh. Lancet. 1987 Sep 26;2(8561):725-8.
15. Vella V, Tomkins A, Ndiku J, Marshal T, Cortinovis I. Anthropometry as a predictor for mortality among Ugandan children, allowing for socio-economic variables. Eur J Clin Nutr. 1994 Mar;48(3):189-97.
16. Caulfield LE, de Onis M, Blossner M, Black RE. Undernutrition as an underlying cause of child deaths associated with diarrhea, pneumonia, malaria, and measles. Am J Clin Nutr. 2004 Jul;80(1):193-8.
17. Scrimshaw NS, Taylor CE, Gordon JE. Interactions of nutrition and infection. Monogr Ser World Health Organ. 1968;57:3-329.
18. Tomkins AM. Nutrition, Infection and Immunity: Public Health Implications. In: Calder PC, Field J, C., Gill HS, editors. Nutrition and Immune Function. Oxford: CAB International; 2002.

19. Cunningham-Rundles S, McNeeley DF, Moon A. Mechanisms of nutrient modulation of the immune response. J Allergy Clin Immunol. 2005 Jun;115(6):1119-28.
20. Bhutta ZA. Effect of infections and environmental factors on growth and nutritional status in developing countries. J Pediatr Gastroenterol Nutr. 2006 Dec;43 Suppl 3:S13-21.
21. Black RE. Would control of childhood infectious diseases reduce malnutrition? Acta Paediatr Scand Suppl. 1991;374:133-40.
22. Ashworth A, Chopra M, McCoy D, Sanders D, Jackson D, Karaolis N, et al. WHO guidelines for management of severe malnutrition in rural South African hospitals: effect on case fatality and the influence of operational factors. Lancet. 2004 Apr 3;363(9415):1110-5.
23. Deen JL, Funk M, Guevara VC, Saloojee H, Doe JY, Palmer A, et al. Implementation of WHO guidelines on management of severe malnutrition in hospitals in Africa. Bull World Health Organ. 2003;81(4):237-43.
24. Maitland K, Berkley JA, Shebbe M, Peshu N, English M, Newton CR. Children with severe malnutrition: can those at highest risk of death be identified with the WHO protocol? PLoS Med. 2006 Dec;3(12):e500.
25. Bachou H, Tumwine JK, Mwadime RK, Tylleskar T. Risk factors in hospital deaths in severely malnourished children in Kampala, Uganda. BMC Pediatr. 2006;6:7.
26. Heikens GT. How can we improve the care of severely malnourished children in Africa? PLoS Med. 2007 Feb;4(2):e45.
27. Berkley JA, Ross A, Mwangi I, Osier FH, Mohammed M, Shebbe M, et al. Prognostic indicators of early and late death in children admitted to district hospital in Kenya: cohort study. Bmj. 2003 Feb 15;326(7385):361.
28. Berkowitz FE. Bacteremia in hospitalized black South African children. A one-year study emphasizing nosocomial bacteremia and bacteremia in severely malnourished children. Am J Dis Child. 1984 Jun;138(6):551-6.
29. Cotton MF, Berkowitz FE, Berkowitz Z, Becker PJ, Heney C. Nosocomial infections in black South African children. Pediatr Infect Dis J. 1989 Oct;8(10):676-83.
30. Isaack H, Mbise RL, Hirji KF. Nosocomial bacterial infections among children with severe protein energy malnutrition. East Afr Med J. 1992 Aug;69(8):433-6.
31. Islam MA, Rahman MM, Mahalanabis D, Rahman AK. Death in a diarrhoeal cohort of infants and young children soon after discharge from hospital: risk factors and causes by verbal autopsy. J Trop Pediatr. 1996 Dec;42(6):342-7.
32. Stephensen CB. Burden of infection on growth failure. J Nutr. 1999 Feb;129(2S Suppl):534S-8S.
33. van Roosmalen-Wiebenga MW, Kusin JA, de With C. Nutrition rehabilitation in hospital--a waste of time and money? Evaluation of nutrition rehabilitation in a rural district hospital in South-west Tanzania. II. Long-term results. J Trop Pediatr. 1987 Feb;33(1):24-8.
34. Hennart P, Beghin D, Bossuyt M. Long-term follow-up of severe protein-energy malnutrition in Eastern Zaire. J Trop Pediatr. 1987 Feb;33(1):10-2.
35. Chapko MK, Prual A, Gamatie Y, Maazou AA. Randomized clinical trial comparing hospital to ambulatory rehabilitation of malnourished children in Niger. J Trop Pediatr. 1994 Aug;40(4):225-30.
36. Ashworth A. WHO Technical Background Paper: Efficacy and effectiveness of community-based treatment of severe malnutrition. Geneva: WHO; 2006.
37. Garrow JS, Pike MC. The long-term prognosis of severe infantile malnutrition. Lancet. 1967 Jan 7;1(7480):1-4.

38. Campbell DI, Elia M, Lunn PG. Growth faltering in rural Gambian infants is associated with impaired small intestinal barrier function, leading to endotoxemia and systemic inflammation. J Nutr. 2003 May;133(5):1332-8.
39. Heikens GT, Schofield WN, Dawson SM, Waterlow JC. Long-stay versus short-stay hospital treatment of children suffering from severe protein-energy malnutrition. Eur J Clin Nutr. 1994 Dec;48(12):873-82.
40. Wagstaff A, van Doorslaer E. Catastrophe and impoverishment in paying for health care: with applications to Vietnam 1993-1998. Health Econ. 2003 Nov;12(11):921-34.
41. Castillo-Duran C, Vial P, Uauy R. Oral copper supplementation: effect on copper and zinc balance during acute gastroenteritis in infants. Am J Clin Nutr. 1990 Jun;51(6):1088-92.
42. Mitra AK, Wahed MA, Chowdhury AK, Stephensen CB. Urinary retinol excretion in children with acute watery diarrhoea. J Health Popul Nutr. 2002 Mar;20(1):12-7.
43. Manary MJ, Broadhead RL, Yarasheski KE. Whole-body protein kinetics in marasmus and kwashiorkor during acute infection. Am J Clin Nutr. 1998 Jun;67(6):1205-9.
44. Manary MJ, Yarasheski KE, Berger R, Abrams ET, Hart CA, Broadhead RL. Whole-body leucine kinetics and the acute phase response during acute infection in marasmic Malawian children. Pediatr Res. 2004 Jun;55(6):940-6.
45. Tomkins AM, Garlick PJ, Schofield WN, Waterlow JC. The combined effects of infection and malnutrition on protein metabolism in children. Clin Sci (Lond). 1983 Sep;65(3):313-24.
46. Waterlow JC, Golden J, Picou D. The measurements of rates of protein turnover, synthesis, and breakdown in man and the effects of nutritional status and surgical injury. Am J Clin Nutr. 1977 Aug;30(8):1333-9.
47. Saez-Llorens X, Lagrutta F. The acute phase host reaction during bacterial infection and its clinical impact in children. Pediatr Infect Dis J. 1993 Jan;12(1):83-7.
48. Benhariz M, Goulet O, Salas J, Colomb V, Ricour C. Energy cost of fever in children on total parenteral nutrition. Clin Nutr. 1997 Oct;16(5):251-5.
49. Du Bois EF. The basal metabolism of fever. Jama. 1921;77 352–5.
50. Stettler N, Schutz Y, Whitehead R, Jequier E. Effect of malaria and fever on energy metabolism in Gambian children. Pediatr Res. 1992 Feb;31(2):102-6.
51. Zaman K, Baqui AH, Yunus M, Sack RB, Bateman OM, Chowdhury HR, et al. Association between nutritional status, cell-mediated immune status and acute lower respiratory infections in Bangladeshi children. Eur J Clin Nutr. 1996 May;50(5):309-14.
52. Zaman K, Baqui AH, Yunus M, Sack RB, Chowdhury HR, Black RE. Malnutrition, cell-mediated immune deficiency and acute upper respiratory infections in rural Bangladeshi children. Acta Paediatr. 1997 Sep;86(9):923-7.
53. Lindtjorn B, Alemu T, Bjorvatn B. Nutritional status and risk of infection among Ethiopian children. J Trop Pediatr. 1993 Apr;39(2):76-82.
54. Pickering H, Hayes RJ, Tomkins AM, Carson D, Dunn DT. Alternative measures of diarrhoeal morbidity and their association with social and environmental factors in urban children in The Gambia. Trans R Soc Trop Med Hyg. 1987;81(5):853-9.
55. Hughes WT, Price RA, Sisko F, Havron WS, Kafatos AG, Schonland M, et al. Protein-calorie malnutrition. A host determinant for Pneumocystis carinii infection. Am J Dis Child. 1974 Jul;128(1):44-52.
56. Russian DA, Levine SJ. Pneumocystis carinii pneumonia in patients without HIV infection. Am J Med Sci. 2001 Jan;321(1):56-65.
57. Tomashefski JF, Jr., Butler T, Islam M. Histopathology and etiology of childhood pneumonia: an autopsy study of 93 patients in Bangladesh. Pathology. 1989 Apr;21(2):71-8.

58. Purtilo DT, Connor DH. Fatal infections in protein-calorie malnourished children with thymolymphatic atrophy. Arch Dis Child. 1975 Feb;50(2):149-52.
59. Chandra RK, Wadhwa M. Nutritional modulation of intestinal mucosal immunity. Immunol Invest. 1989 Jan-May;18(1-4):119-26.
60. Sirisinha S, Suskind R, Edelman R, Asvapaka C, Olson RE. Secretory and serum IgA in children with protein-calorie malnutrition. Pediatrics. 1975 Feb;55(2):166-70.
61. Reddy V, Raghuramulu N, Bhaskaram C. Secretory IgA in protein-calorie malnutrition. Arch Dis Child. 1976 Nov;51(11):871-4.
62. Gilman RH, Partanen R, Brown KH, Spira WM, Khanam S, Greenberg B, et al. Decreased gastric acid secretion and bacterial colonization of the stomach in severely malnourished Bangladeshi children. Gastroenterology. 1988 Jun;94(6):1308-14.
63. Martinsen TC, Bergh K, Waldum HL. Gastric juice: a barrier against infectious diseases. Basic Clin Pharmacol Toxicol. 2005 Feb;96(2):94-102.
64. Chevalier P, Sevilla R, Sejas E, Zalles L, Belmonte G, Parent G. Immune recovery of malnourished children takes longer than nutritional recovery: implications for treatment and discharge. J Trop Pediatr. 1998 Oct;44(5):304-7.
65. Parent G, Chevalier P, Zalles L, Sevilla R, Bustos M, Dhenin JM, et al. In vitro lymphocyte-differentiating effects of thymulin (Zn-FTS) on lymphocyte subpopulations of severely malnourished children. Am J Clin Nutr. 1994 Aug;60(2):274-8.
66. Smythe PM, Brereton-Stiles GG, Grace HJ, Mafoyane A, Schonland M, Coovadia HM, et al. Thymolymphatic deficiency and depression of cell-mediated immunity in protein-calorie malnutrition. Lancet. 1971 Oct 30;2(7731):939-43.
67. Najera O, Gonzalez C, Toledo G, Lopez L, Ortiz R. Flow cytometry study of lymphocyte subsets in malnourished and well-nourished children with bacterial infections. Clin Diagn Lab Immunol. 2004 May;11(3):577-80.
68. Najera O, Gonzalez C, Toledo G, Lopez L, Cortes E, Betancourt M, et al. CD45RA and CD45RO isoforms in infected malnourished and infected well-nourished children. Clin Exp Immunol. 2001 Dec;126(3):461-5.
69. Fakhir S, Ahmad P, Faridi MA, Rattan A. Cell-mediated immune responses in malnourished host. J Trop Pediatr. 1989 Aug;35(4):175-8.
70. Rodriguez L, Graniel J, Ortiz R. Effect of leptin on activation and cytokine synthesis in peripheral blood lymphocytes of malnourished infected children. Clin Exp Immunol. 2007 Mar 9.
71. Ortiz R, Campos C, Gomez JL, Espinoza M, Ramos-Motilla M, Betancourt M. Effect of renutrition on the proliferation kinetics of PHA stimulated lymphocytes from malnourished children. Mutat Res. 1995 Apr;334(2):235-41.
72. Hughes S, Kelly P. Interactions of malnutrition and immune impairment, with specific reference to immunity against parasites. Parasite Immunol. 2006 Nov;28(11):577-88.
73. Gotch FM, Spry CJ, Mowat AG, Beeson PB, Maclennan IC. Reversible granulocyte killing defect in anorexia nervosa. Clin Exp Immunol. 1975 Aug;21(2):244-9.
74. Jose DG, Shelton M, Tauro GP, Belbin R, Hosking CS. Deficiency of immunological and phagocytic function in aboriginal children with protein-calorie malnutrition. Med J Aust. 1975 Nov 1;2(18):699-705.
75. McMurray DN, Watson RR, Reyes MA. Effect of renutrition on humoral and cell-mediated immunity in severely malnourished children. Am J Clin Nutr. 1981 Oct;34(10):2117-26.
76. Chevalier P, Sevilla R, Zalles L, Sejas E, Belmonte G, Parent G, et al. [Immuno-nutritional recovery of children with severe malnutrition]. Sante. 1996 Jul-Aug;6(4):201-8.
77. Geefhuysen J, Rosen EU, Katz J, Ipp T, Metz J. Impaired cellular immunity in kwashiorkor with improvement after therapy. Br Med J. 1971 Nov 27;4(5786):527-9.

78. Parent MA, Loening WE, Coovadia HM, Smythe PM. Pattern of biochemical and immune recovery in protein calorie malnutrition. S Afr Med J. 1974 Jul 6;48(32):1375-8.
79. Ford GW, Belbin R, Jose DG, Vorbach EA, Kirke DK. Growth and immune function in Aboriginal children during recovery from malnutrition and infection. Aust N Z J Med. 1976 Aug;6(4):321-8.
80. Chintu C, Bhat GJ, Walker AS, Mulenga V, Sinyinza F, Lishimpi K, et al. Co-trimoxazole as prophylaxis against opportunistic infections in HIV-infected Zambian children (CHAP): a double-blind randomised placebo-controlled trial. Lancet. 2004 Nov 20-26;364(9448):1865-71.
81. Mulenga V, Ford D, Walker AS, Mwenya D, Mwansa J, Sinyinza F, et al. Effect of cotrimoxazole on causes of death, hospital admissions and antibiotic use in HIV-infected children. Aids. 2007 Jan 2;21(1):77-84.
82. Mermin J, Lule J, Ekwaru JP, Malamba S, Downing R, Ransom R, et al. Effect of co-trimoxazole prophylaxis on morbidity, mortality, CD4-cell count, and viral load in HIV infection in rural Uganda. Lancet. 2004 Oct 16-22;364(9443):1428-34.
83. Grimwade K, Sturm AW, Nunn AJ, Mbatha D, Zungu D, Gilks CF. Effectiveness of cotrimoxazole prophylaxis on mortality in adults with tuberculosis in rural South Africa. Aids. 2005 Jan 28;19(2):163-8.
84. Castetbon K, Anglaret X, Attia A, Toure S, Dakoury-Dogbo N, Messou E, et al. Effect of early chemoprophylaxis with co-trimoxazole on nutritional status evolution in HIV-1-infected adults in Abidjan, Cote d'Ivoire. Aids. 2001 May 4;15(7):869-76.
85. Wiktor SZ, Sassan-Morokro M, Grant AD, Abouya L, Karon JM, Maurice C, et al. Efficacy of trimethoprim-sulphamethoxazole prophylaxis to decrease morbidity and mortality in HIV-1-infected patients with tuberculosis in Abidjan, Cote d'Ivoire: a randomised controlled trial. Lancet. 1999 May 1;353(9163):1469-75.
86. WHO. Pocket book for hospital care of children: guidelines for the management of common illness with limited resources. 2005.
87. Garly ML, Bale C, Martins CL, Whittle HC, Nielsen J, Lisse IM, et al. Prophylactic antibiotics to prevent pneumonia and other complications after measles: community based randomised double blind placebo controlled trial in Guinea-Bissau. Bmj. 2006 Dec 16;333(7581):1245.
88. Cale CM, Jones AM, Goldblatt D. Follow up of patients with chronic granulomatous disease diagnosed since 1990. Clin Exp Immunol. 2000 May;120(2):351-5.
89. Mouy R, Fischer A, Vilmer E, Seger R, Griscelli C. Incidence, severity, and prevention of infections in chronic granulomatous disease. J Pediatr. 1989 Apr;114(4 Pt 1):555-60.
90. Gurwith M, Truog K, Hinthorn D, Liu C. Trimethoprim-sulfamethoxazole and trimethoprim alone for prophylaxis of infection in granulocytopenic patients. Rev Infect Dis. 1982 Mar-Apr;4(2):593-601.
91. Goldblatt D. Current treatment options for chronic granulomatous disease. Expert Opin Pharmacother. 2002 Jul;3(7):857-63.
92. Walker AS, Mulenga V, Sinyinza F, Lishimpi K, Nunn A, Chintu C, et al. Determinants of survival without antiretroviral therapy after infancy in HIV-1-infected Zambian children in the CHAP Trial. J Acquir Immune Defic Syndr. 2006 Aug 15;42(5):637-45.
93. Mermin J, Lule J, Ekwaru JP, Downing R, Hughes P, Bunnell R, et al. Cotrimoxazole prophylaxis by HIV-infected persons in Uganda reduces morbidity and mortality among HIV-uninfected family members. Aids. 2005 Jul 1;19(10):1035-42.
94. Thera MA, Sehdev PS, Coulibaly D, Traore K, Garba MN, Cissoko Y, et al. Impact of trimethoprim-sulfamethoxazole prophylaxis on falciparum malaria infection and disease. J Infect Dis. 2005 Nov 15;192(10):1823-9.

95. Rozin A, Schapira D, Braun-Moscovici Y, Nahir AM. Cotrimoxazole treatment for rheumatoid arthritis. Semin Arthritis Rheum. 2001 Oct;31(2):133-41.
96. Gmunder FK, Seger RA. Chronic granulomatous disease: mode of action of sulfamethoxazole/trimethoprim. Pediatr Res. 1981 Dec;15(12):1533-7.
97. Climax J, Lenehan TJ, Lambe R, Kenny M, Caffrey E, Darragh A. Interaction of antimicrobial agents with human peripheral blood leucocytes: uptake and intracellular localization of certain sulphonamides and trimethoprims. J Antimicrob Chemother. 1986 Apr;17(4):489-98.
98. Carre P, Forgue MF, Pipy B, Beraud M, Bessieres MH, Didier A, et al. [Effects of cotrimoxazole on some macrophage functions: microbicide, tumoricide, production of free oxygen radicals, prostaglandins and leukotrienes]. Pathol Biol (Paris). 1990 Apr;38(4):289-93.
99. Kobayashi M, Sato T, Kawaguchi H, Nakamura K, Kihara H, Hiraoka A, et al. Efficacy of prophylactic use of trimethoprim-sulfamethoxazole in autoimmune neutropenia in infancy. J Pediatr Hematol Oncol. 2003 Jul;25(7):553-7.
100. Grimwade K, Swingler. Cotrimoxazole prophylaxis for opportunistic infections in adults with HIV. Cochrane Database Syst Rev. 2003(3):CD003108.
101. WHO. Guidelines on co-trimoxazole prophylaxis for HIV-related infections among children, adolescents and adults: Recommendations for a public health approach. 2006.
102. Zar HJ, Hanslo D, Hussey G. The impact of HIV infection and trimethoprim-sulphamethoxazole prophylaxis on bacterial isolates from children with community-acquired pneumonia in South Africa. J Trop Pediatr. 2003 Apr;49(2):78-83.
103. Polack FP, Flayhart DC, Zahurak ML, Dick JD, Willoughby RE. Colonization by Streptococcus penumoniae in human immunodeficiency virus-infected children. Pediatr Infect Dis J. 2000 Jul;19(7):608-12.
104. Abdel-Haq N, Abuhammour W, Asmar B, Thomas R, Dabbagh S, Gonzalez R. Nasopharyngeal colonization with Streptococcus pneumoniae in children receiving trimethoprim-sulfamethoxazole prophylaxis. Pediatr Infect Dis J. 1999 Jul;18(7):647-9.
105. Jordano Q, Falco V, Almirante B, Planes AM, del Valle O, Ribera E, et al. Invasive pneumococcal disease in patients infected with HIV: still a threat in the era of highly active antiretroviral therapy. Clin Infect Dis. 2004 Jun 1;38(11):1623-8.
106. Madhi SA, Petersen K, Madhi A, Wasas A, Klugman KP. Impact of human immunodeficiency virus type 1 on the disease spectrum of Streptococcus pneumoniae in South African children. Pediatr Infect Dis J. 2000 Dec;19(12):1141-7.
107. Zachariah R, Harries AD, Spielmann MP, Arendt V, Nchingula D, Mwenda R, et al. Changes in Escherichia coli resistance to co-trimoxazole in tuberculosis patients and in relation to co-trimoxazole prophylaxis in Thyolo, Malawi. Trans R Soc Trop Med Hyg. 2002 Mar-Apr;96(2):202-4.
108. Watera C, Todd J, Muwonge R, Whitworth J, Nakiyingi-Miiro J, Brink A, et al. Feasibility and effectiveness of cotrimoxazole prophylaxis for HIV-1-infected adults attending an HIV/AIDS clinic in Uganda. J Acquir Immune Defic Syndr. 2006 Jul;42(3):373-8.
109. Martin JN, Rose DA, Hadley WK, Perdreau-Remington F, Lam PK, Gerberding JL. Emergence of trimethoprim-sulfamethoxazole resistance in the AIDS era. J Infect Dis. 1999 Dec;180(6):1809-18.
110. Iyer JK, Milhous WK, Cortese JF, Kublin JG, Plowe CV. Plasmodium falciparum cross-resistance between trimethoprim and pyrimethamine. Lancet. 2001 Sep 29;358(9287):1066-7.
111. McNally LM, Jeena PM, Gajee K, Sturm AW, Tomkins AM, Coovadia HM, et al. Lack of Association between the Nasopharyngeal Carriage of Streptococcus pneumoniae and Staphylococcus aureus in HIV-1-Infected South African Children. J Infect Dis. 2006 Aug 1;194(3):385-90.

112. Jelinek T, Kilian AH, Curtis J, Duraisingh MT, Kabagambe G, von Sonnenburg F, et al. Plasmodium falciparum: selection of serine 108 of dihydrofolate reductase during treatment of uncomplicated malaria with co-trimoxazole in Ugandan children. Am J Trop Med Hyg. 1999 Jul;61(1):125-30.
113. Malamba SS, Mermin J, Reingold A, Lule JR, Downing R, Ransom R, et al. Effect of cotrimoxazole prophylaxis taken by human immunodeficiency virus (HIV)-infected persons on the selection of sulfadoxine-pyrimethamine-resistant malaria parasites among HIV-uninfected household members. Am J Trop Med Hyg. 2006 Sep;75(3):375-80.
114. Ryan M, Griffin S, Chitah B, Walker AS, Mulenga V, Kalolo D, et al. The cost-effectiveness of cotrimoxazole prophylaxis in HIV-infected children in Zambia. Aids. 2008 Mar 30;22(6):749-57.
115. Goldie SJ, Yazdanpanah Y, Losina E, Weinstein MC, Anglaret X, Walensky RP, et al. Cost-effectiveness of HIV treatment in resource-poor settings--the case of Cote d'Ivoire. The New England journal of medicine. 2006 Sep 14;355(11):1141-53.
116. Pitter C, Kahn JG, Marseille E, Lule JR, McFarland DA, Ekwaru JP, et al. Cost-effectiveness of cotrimoxazole prophylaxis among persons with HIV in Uganda. J Acquir Immune Defic Syndr. 2007 Mar 1;44(3):336-43.
117. Nzila AM, Mberu EK, Sulo J, Dayo H, Winstanley PA, Sibley CH, et al. Towards an understanding of the mechanism of pyrimethamine-sulfadoxine resistance in Plasmodium falciparum: genotyping of dihydrofolate reductase and dihydropteroate synthase of Kenyan parasites. Antimicrobial agents and chemotherapy. 2000 Apr;44(4):991-6.
118. Nzila AM, Nduati E, Mberu EK, Hopkins Sibley C, Monks SA, Winstanley PA, et al. Molecular evidence of greater selective pressure for drug resistance exerted by the long-acting antifolate Pyrimethamine/Sulfadoxine compared with the shorter-acting chlorproguanil/dapsone on Kenyan Plasmodium falciparum. J Infect Dis. 2000 Jun;181(6):2023-8.

25. INFORMED CONSENT INFORMATION AND FORMS

CTX STUDY

Investigators:

Dr James Berkley (KEMRI Kilifi)

Johnstone Thitiri (KEMRI Kilifi)

Dr Greg Fegan (KEMRI Kilifi)

Dr Twahir Hemed (CPGH, Mombasa)

Dr Victor Bandika (CPGH, Mombasa)

Dr Maurice Buni (MDH, Malindi)

Dr Florence Kapanga (MDH, Malindi)

Ms Laura Mwakela (KEMRI Kilifi)

Mrs Fauzat Hamid (KEMRI Kilifi)

Ms Rehema Ali (KEMRI Kilifi)

Dr Judd Walson (KEMRI, Nairobi)

Dr Kathryn Maitland (KEMRI Kilifi)

Dr Roma Chilengi (KEMRI Kilifi)

What is KEMRI?

KEMRI is part of the Ministry of Health that carries out research with the aim of finding better ways of prevention and treating illness in the future, for everybody's benefit. We are asking your permission for your child to participate in a study.

What is this research about?

Your child has been admitted to this hospital and found to have malnutrition. Malnutrition is known to place children at risk getting diseases like malaria, pneumonia or diarrhoea, even after discharge from the hospital.

A common drug called 'cotrimoxazole' or 'septrin' is used to treat wide range of infections especially of the chest. This study aims to determine if a small dose of cotrimoxazole given every day for six months can prevent such infections and improve the life of children with malnutrition.

In order to find out whether cotrimoxazole is effective for this purpose, half the children in this study will receive cotrimoxazole whilst the other half will receive a dummy drug that looks the same, but has no cotrimoxazole in it. The decision on which child will get the drug or not will be by chance and neither you or any of our study staff will know the treatment your child is in, until the end of the study.

What will it involve for me/my child?

1. Your child will continue to receive usual care and treatment for the condition with which he/she is admitted until discharge.
2. Once your child has become stable on the hospital treatment a doctor will prescribe the study drug in form of a tablet that dissolves in water, to be taken once a day.
3. At discharge you will be provided with the drug every month for a total of six months. You are required to visit our clinic once a month for your child to be weighed and measured and receive more drugs. Fares to attend these appointments will be provided to you.
4. We will take small blood samples (5ml, a teaspoon full) from your child now, at 2, 6 and 12 months into this study. We will also perform throat swabs and faecal samples at these times to check for common infections there.
5. If your child becomes sick during the study, we would like you to bring your child at the paediatric ward or study clinic. We will pay the cost of treatment available at this hospital.
6. After the study drugs are finished after 6 months, we will ask you to come back every two months until one year is over. At this time your child will have completed the study.

<u>Are there any risks from my child's participation?</u>

The study drug, cotrimoxazole is widely used in Kenya, however, as with any drug side effects can sometimes occur. Very rarely, the drug may affect the skin or blood and we ask you to report these to us immediately. Some pain and discomfort will be experienced from the needle prick when blood samples are obtained. Careful attention will however be paid to ensure that the sample is taken in a professional manner.

<u>Are there any benefits for my/my child's participation into this study?</u>

The benefits for your child taking part are that your child will get close observation during the trial. In addition your child will receive free consultation and treatment during the time of the study. The results of this study will be beneficial to society at large as they may influence how we care for children with malnutrition.

<u>What will happen if I do not agree to participate?</u>

Participation in research is voluntary. You are free to decide if you want your child to take part in research or not. Your child will still receive the recommended standard care whether or not you agree to take part. If you have decided to participate, you can change your mind any time and withdraw your child from the study. This will not affect your child's care now or in the future.

<u>What will happen to the blood and other samples?</u>

After this research a small volume of blood, stool and throat swab will be stored for testing how the body is working against infections. Some of these tests will be done overseas if they are not available in Kenya, if you permit us to do that.

<u>Who will be involved in accessing my child's information in this study?</u>

All information on participants collected in this study will be stored in a confidential manner in locked, secured cabinets and password protected computers and will only be accessible to

authorised study personnel. Data will be stored to the end of the study and analysis. Any report or publications on this study will not use participant's names or identities.

Who has authorized this research to take place?

The independent National Research and Ethics Committee in Nairobi; the Scientific Steering Committee in Kilifi; and a committee in Oxford University in the UK have checked and approved this work.

What if I have any questions?

You can ask any of our workers anytime. You can also communicate with those involved in taking care of your child and this study.

or

Dr. Jay Berkley KEMRI-Wellcome Trust, PO Box 230, Kilifi Kenya Tel no. 041 7522 063

Johnstone Thitiri KEMRI-Wellcome Trust, PO Box 230, Kilifi Kenya Tel. no. 041 7522 063

If you would like to communicate with someone not involved in this study please contact: The Community Liaison Office hotline number 0723 342 870 between 8am and 5pm. or

The Secretary, KEMRI Ethics Committee,

P.O Box 54840-00200. Nairobi.

Telephone no. 020 272 2541. Mobile 0722205901 or 0733400003.

CTX Study (Kiswahili)

Watafiti

Dr James Berkley (KEMRI Kilifi)

Johnstone Thitiri (KEMRI Kilifi)

Dr Greg Fegan (KEMRIKilifi)

Dr Twahir Hemed (Paediatrician, CPGH,Mombasa)

Dr Maurice Buni (DMoH,Malindi)

DR Judd Walson (KEMRI Nairobi)

Dr KathrynMaitland (KEMRI Kilifi)

Dr Roma Chilengi (KEMRI Kilifi)

Je, KEMRI ni nini?

KEMRI ni shirika la serikali ambalo linafanya utafiti wa kimadawa kutafuta njia bora za kuzuia na kutibu magonjwa kwa siku za usoni kwa manufaa ya kila mtu. Tunakuomba ruhusa kumshirikisha mtoto wako katika utafiti huu.

Mradi huu unahusu nini?

Mtoto wako amelazwa katika hospitali hii na kupatikana kuwa na ukosefu wa lishe bora. Matibabu ya ukosefu wa lishe bora na magonjwa mengine yameanzishwa tayari. Ukosefu wa lishe bora inajulikana kuweka watoto katika hatari ya kupata magonjwa kama malaria, homa ya mapafu, au kuharisha, hata baada ya kupata kibali kwenda nyumbani baada ya kulazwa hospitalini. Tunatafuta mbinu ya kupunguza hatari hii.

KEMRI kwa sasa inaendesha uchunguzi ili kutambua kama dawa ya kawaida iitwayo Cotrimoxazole au "Septrin" inaweza kupunguza hatari hii. Cotrimoxazole inatumika kwa sasa kutibu maambukizo ya aina nyingi kwa watoto na hata watu wazima ,hasa yale kifua. Uchunguzi huu unanuia kubainisha kama kipimo kidogo cha Cotrimoxazole kikitumiwa mara moja kila siku kwa muda wa miezi sita yaweza kuzuia mangonjwa kama haya na kuboresha maisha ya watoto walio na ukosefu wa lishe bora. Katika utafiti huu, tunanuia kujumlisha watoto 1600 walio na ukosefu wa lishe bora.

Ili kubainisha kama dawa ya cotrimoxazole ina uwezo mzuri katika kazi hii nusu ya watoto katika mradi huu watapata dawa ya cotrimoxazole ilihali nusu ile nyingine watapata dawa bonzo ambayo yafanana sawa na yakwanza lakini haina cotrimoxazole ndani yake. Dawa hii ya kuiga haitakuwa na kemikali ya dawa ya cotrimoxazole bali tu viungo vya ladha. Uamuzi wa kutambua ni yupi kati ya watoto atapata dawa ya cotrimoxazole au ile ya kuiga itafanyika kwa mpangilio wa bahati nasibu na hakuna mtu kati ya wewe au yeyote kati ya watafiti katika mradi watajua matibabu yapi mtoto wako atapata, hadi mwisho wa mradi. Tutaweza kutambua kama dawa ya cotrimoxazole inawasaidia watoto kwa kulinganisha kuendelea kwa watoto watakaotumia dawa hii na wale ambao watatumia dawa bonzo ya kuiga katika huo muda wa miezi sita.

Ni nini kitafanyika kwangu/mtoto wangu?

1. Mtoto wako ataendelea kupata matibabu ya kawaida ya maradhi yaliyomfanya alazwe hospitalini hadi wakati wa kupata ruhusa kurudi nyumbani.
2. Mara mtoto wako atakapoimarika na kupata nafuu kutokana na matibabu hospitalini, daktari atamwanzishia dawa ya uchunguzi ambayo ni tembe inayoyeyuka kwa maji, ya kumezwa mara moja kwa siku, kila siku kwa muda wa miezi sita, pamoja na muda ambapo mtoto atakuwa amerudi nyumbani.
3. Wakati wa kuruhusiwa kurudi nyumbani, utaulizwa kurudi katika kliniki yetu kila mwezi kwa muda wa miezi sita, halafu kila baada ya miezi miwili baadaye hadi mwaka mmoja umalizike. Katika kila kliniki unapokuja katika muda wa kwanza wa miezi sita, utapewa dawa ya kutosha kumaliza mwezi mmoja.Pia mtoto wako atapimwa uzito na vipimo vingine. Utapewa pesa ya kugharamia nauli ya kuhudhuria kliniki hizi.
4. Tutachukua sampuli kidogo ya damu ya 5 ml (kama kijiko kimoja kidogo cha chai) kutoka kwa mtoto wako sasa, katika mwezi wa tatu na pia wa sita anavyoendelea katika huu mradi. Pia tutachukua kiwango cha kikohozi kutoka kwa koo la mtoto na pia sampuli ya choo cha mtoto katika muda huu pia ili kutumia kuchunguza mangonjwa ya kawaida.
5. Ikiwa mtoto wako atakuwa mgonjwa wakati wa utafiti, tungependa umlete katika wodi ya kulaza watoto ya hii hospitali au katika kliniki yetu ya mradi. Tutalipa gharama ya matibabu yake katika hii hospitali.

Je, kuna hatari yeyote kutokana na kushiriki kwa mtoto wangu?

Dawa inayotumika kwa uchunguzi ya cotrimoxazole imesajiliwa kwa matumizi kwa watoto na hata watu wazima kote ulimwenguni, na pia inatumika kwa mapana hapa Kenya. Hata hivyo, kama ilivyo kawaida na dawa yeyote, madhara fulani yawezatokea wakati mwingine. Kwa nadra sana, dawa hii yaweza kudhuru ngozi na damu na hivyo tunakuomba kutujulisha shida yeyote ya ngozi, vijidonda vya mdomo au magonjwa yeyote kwa dharura. Ikiwa tutapata habari mpya kuhusu Cotrimoxazole tutakueleza wewe. Uchungu kiasi wa muda mfupi utatokea kwa mtoto wako kutokana na kudungwa sindano wakati wa kutoa sampuli ya damu. Sampuli za damu hutolewa tu na wafanyakazi wa KEMRI ambao wamepata mafunzo ya kufanya kazi hii kwa usalama.

Je, kuna manufaa kwangu/ au mtoto wangu kwa kushiriki katika mradi huu?

Manufaa ya mtoto wako kushiriki ni kwamba, mtoto wako atapata matibabu ya kiafya wakati wote alipo katika uchunguzi huu kwa njia ya kuonekana na daktari na kupata matibabu ya bure. Matokeo ya uchunguzi huu yatafaidisha jamii kwa ujumla na yanaweza kutupa mwelekeo mpya kuhusu huduma kwa watoto walio na ukosefu wa lishe bora.

Ni nini kitafanyika ikiwa sitakubali kuhusika?

Kuhusika katika utafiti ni hiari. Una uhuru wa kuamua ikiwa unataka mtoto wako ahusike katika utafiti huu au la. Mtoto wako bado atapata usaidizi wa sawa na wale wengine iwapo utakubali au ukatae. Iwapo umeamua kuhusika, unaweza kubadilisha mawazo yako wakati wowote na utoe mtoto wako katika mradi. Hii haitadhuru usaidizi wa mtoto wako sasa na hata baadaye.

Nini kitafanyika kwa damu na hizo sampuli nyingeni?

Baada ya utafiti huu kiasi kidogo cha damu ,kinyesi na sehemu ya kikohozi kutoka koo (throat swab) zitahifadhiwa kwa upimaji wa jinsi mwili unavyofanya kazi dhidi ya maradhi .Baadhi ya vipimo hivi vitafanywa katika nchi ya ng'ambo kama hazipatikani hapa nchini Kenya ikiwa

utaturuhusu kufanya hivyo.utafiti wowote wa siku za usoni wa kutumia sampuli hizi utafanywa tu baada ya kupata thibitisho kutoka (national research and ethic committee)

Ni nani atahusika kupata habari za utafiti wa mtoto wangu?

Habari zote za wahusika zitakazochukuliwa katika huu utafiti zitahifadhiwa kwa njia ya siri katika kabati zilizofungwa na mitambo ya computa iliyowekwa vizuizi na habari hizi zitafikiwa tu na watafiti wa mradi walioruhusiwa. Habari hizi zitahifadhiwa hadi mwisho wa utafiti na baada ya uchambuzi wake. Ripoti yeyote au tahariri kuhusu uchunguzi huu haitatumia majina ya wahusika wala kutambua walioshiriki.

Ni nani ameruhusu huu utafiti kufanywa?

Kamati huru ya kitaifa ya utafiti na maadili, kamati ya kisayansi hapa Kilifi na kamati nyingine ya kisayansi na maadili ya Oxford University Uingereza, zimechunguza kwa makini kazi hii na kukubali kwamba uchunguzi huu ni muhimu na kuwa uko na usalama kushiriki na kuwa utafiti utafanywa kwa njia inayohistahili

Je, kama nina swali lolote?

Unaweza kuuliza maswali mfanyi kazi wetu yeyote wakati wowote. Unaweza pia kuwasiliana na wale ambao wanahusika na matibabu ya mtoto wako na walio katika utafiti huu.

au

Dr Jay Berkley KEMRI- Wellcome Trust P.O.Box. 230, Kenya. Simu: 041 522 063

Ikiwa unataka kuuliza mtu yoyote ambaye hahusiki na utafiti huu tafadhali wasiliana na:

The Community Liaison Office Hotline Number 0723 342870 kati ya saa mbili asubuhi (8am) na saa kumi na moja jioni (5 pm).

au

Mwandishi (Secretary) – KEMRI Ethics Committee -(Kamati ya maadili ya kitaifa.)

S.L.P. 54840-00200, Nairobi. Nambari ya simu: 020 272 2541 Simu ya mkono: 0722205901 au 0733400003

CTX STUDY (Kigiriama)

Atafiti:

Dr James Berkley (KEMRI Kilifi)

Johnstone Thitiri (KEMRI Kilifi)

Dr Greg Fegan (KEMRIKilifi)

Dr Twahir Hemed (Paediatrician, CPGH,Mombasa)

Dr Maurice Buni (DMoH,Malindi)

DR Judd Walson (KEMRI Nairobi)

Dr Kathryn Maitland (KEMRI Kilifi)

Dr Roma Chilengi (KEMRI Kilifi)

Je KEMRI ni noni?

KEMRI ni sehemu ya wizara ya afya ambayo inahenda utafiti kwa lengo ra kumala ngira mbidzo za kuzulia makongo kwa siku zidzazo,kwa manufaa ga kila mtu. Funakuvoya ruhusa ili mwanao ahusike kahiza mradhi uu.

Je utafiti uno unahusu noni?

Mwanao adzalazwa kahiza sipitali ii na anaonekana kukala ana upungufu wa chakurya bora. Upungufu wa chakurya bora unamanyikana kukala inaahenda ahoho makale kahiza hathari ya kupata makongo here malaria, pneumonia na kumwaga,hatha baada ya kumbola sipitali baada ya kulazwa.

Dawa ya kawaida iifwayo 'Cotrimoxazole' hedu 'septrin' inatumiwa kutibu makongo manji kwa mfano makongo ga kifuwa. Mradhi uu unalenga kubainisha kala kiwango kichache cha dawa ya Cotrimoxazole ikilazwa kila siku kwa miezi mihandahu kala inadima kuzulia maradhi gaga na kuimarisha maisha ga ahoho enye upungufu wa lishe bora.

Ili kudima kumanya kala Cotrimoxazole ni dawa mbidzo kwa shida ii. Nusu ya ahoho kahiza mradhi uu mandapata Cotrimoxazole na yo nusu nyingine mandapewa dawa nyingine ambayo ni here iyo, lakini kaina cotrimoxazole ndani ya kwakwe. Uamuzi wa kukala mwanao andapata dawa ama kandapata undaamuliwa kwa ngira ya bahathi nasibu na uwe ama yoyosi kahiza ahendzi a kazi a mradhi uu kamandamanya kala matibabu gani mwanao apewago,paka mwisho wa mradhi.

Je indahusu noni kwangu ama kwa mwanangu akihusika kahiza mradhi uno?

1. Mwanao andaenderera kupata kutsunzwa na matibabu ga kawaida kwa yo shidha ambayo mwanao andakala adzalazirwa paka ambozwe.
2. Wakathi thu mwanao andekala ana kabaha kumbolerana na matibabu andekala adzagapata haha sipitali dakithari andamudhikira dawa ya mradhi ambazo ni tembe ambazo zinamumunyika kwenye madzi, zihumirwe lumwenga kwa siku.
3. Baada ya kumbola,undapewa dawa zizi kila mwezi kwa muda wa miezi mihandahu. Unahendzekana udze kiliniki hehu kila mwezi ili mwanao apimwe uziho na vipimo vingine na apewe madawa zaidhi .Tikiti ya kudza kwa zizi siku za kiliniki undapewa.
4. Fundahala kiwango kidogo cha mlatso (5ml,kijiko cha chai)kumbola kwa mwanao vivi,kahiza mwezi wa hahu na wa handahu kahiza uu mradhi. Kisha fundahala sampuli kahiza mumiro na choo kahiza wakathi uu ili kulola maradhi ga kawaida mumo.

5. Kala mwanao andakala mkongo kahiza wakathi wa mradhi,fungehenza umurehe mwanao kahiza wadi ya ahoho ama kahiza kiliniki ya mradhi. Fundarihira garama ya huduma za matibabu garigo ganapatikana kahiza sipitali ii.
6. Baada ya dawa za mradhi kugoma baada ya miezi mihandahu,fundakwambira udze kila baada ya miezi miri paka mwaka mumwenga ugome.Wakathi uu mwanao andakala adzamarigiza mradhi.

Je kuna madhara gogosi ga mwanangu kuhusika?

Dawa ya mradhi,cotrimoxazole inahumirwa sana haha Kenya, lakini, dawa yoyosi mara nyingine inadima kukala na madhara .Lakini kwa kiwango kichache sana,dawa inadima kudhuru ngozi hedu damu na funakuvoya ufuelezere habari haraka idimikikazho. Maumivu kidogo na kubujika kidogo kundaonekana kuombola kwa shindano wakathi wa kuhala sampuli ya mlatso .Uangalifu wa hali ya dzulu undazingatiwa ili kuhakikisha kukala sampuli inahalwa hali ya kiujuzi.

Je kuna manufaa gogosi kwa mwanangu kuhusika kahiza mradhi uno?

Manufaa ga mwanao kuhusika kahiza mradhi uu ni kukala mwanao andapata uangalizi wa hehi kahiza muda wa jaribio riri. Kungezera, mwanao andaonewa ni dakithari na andapata matibabu ga bule kahiza wakathi wa mradhi. Majibu ga mradhi uu gandafaidhisha jamii nzima kwa sababu ganadima kumboza muelekeo kukirira na viryahu ahoho enye upungufu wa chakurya bora mahudumiwazho.

Je kundakalani nikikahala kuhusika kahiza mradhi uno?

Kuhusika kahiza utafiti ni hiari. Uhuru kuamua kala unahedza kushiriki . Una uhuru kuamua kala unahenza mwanao ahusike kahiza mradhi hedu kwenzi. Mwanao andapata huduma za kawaida ukikubali kuhusika hedu usihokubali kuhusika kahiza mradhi. Kala unzaamua kuhusika unadima ukabadhilisha maazo ga kwako wakathi wowosi na unadima kumumboza mwanao kahiza mradhi. Ii kaindaathiri kupata uangalizi wa huduma kwa mwanao vivi na siku zidzazo.

Je mlatso na na sampuli nyingine zindahenderyadze?

Baada ya utafiti uu,kiwango kidogo cha mlatso, choo na sampuli kumbola mumironi vindaikwa ili kupimwa kulola viryahu mwiri ukabilianazho na makongo ga maambukizi.Vipimo vingine vindahenderwa kuko ngambo kala kavidima kuhendeka haha Kenya mkifuruhusu kuhenda vizho.

Nihani andiyehusika kulola habari za mwanangu kahiza mradhi uno?

Habari zosi zindizohalwa kumbola kwa ahusika zindaikwa kahiza ngira ya siri kahiza kabati zidzizofungwa na namba ya siri na zindaruhusiwa kuonewa thu ni atu madzioruhusiwa mario kahiza mradhi uu.Habari zindaikwa hatha mwisho wa mradhi na mwisho wa kudhika ripoti.Ripoti yoyosi ama uchapishaji wa mradhi uu kaundatumia madzina ga atu.

Ni hani adzeruhusu utafiti uno uhendeke?

Kamati huru ya kitaifa na kamati ya maadili kuko Nairobi ;kamati ya kisayansi Kilifi;na kamati kuko chuo kikuu cha Oxford kuko UK zalola kazi ii na maamua kukala utafiti uu ni muhimu,na usalama kuhendeka na undahendwa tototo.

Je kala nina swali rorosi?

Unadima kumuza muhendi wa kazi wehu yoyosi wakathi wowosi.Unadima kuwasiliana na ao ambao manahusika na kumutsunza mwanao na mradhi uu.

hedu

Dr. Jay Berkley KEMRI-Wellcome Trust, PO BOX 230, Kilifi Kenya namba ya simu. 0417522063.

Johnstone Thitiri KEMRI –Welcome trust, PO BOX 230,Kilifi Kenya Namba ya simu. 041 7522063.

Kala unahenza kuwasiliana na mtu ambaye kahusika na mradhi uu tafadhali wasiliana: Office ya uhusiano mdzo kwa jamii namba ya simu 0723342870 kahi ya saa mbiri za madzacha na saa kumi na mwenga za dziloni.

hedu

Mwadhishi ,Kamati ya maadili KEMRI,PO BOX 54840-00200.Nairobi.

Namba ya simu: 020 272 2541.simu ya mkono: 0722205901 ama 0733400003.

KEMRI-Wellcome Programme Consent Form: CTX Study

I, being the parent/ guardian of --------------------------- (name of the child), have been given the explanation about this study.
I have understood what has been explained to me and my questions have been answered satisfactorily. I understand that I can change my mind any time and it will not affect the management of my child.
Please fill the boxes as required:

☐ Please tick. I have agreed to allow my child to participate
☐ Please tick. I have agreed for samples to be stored
☐ Please tick. I have agreed for samples to be exported for overseas testing.

Parent/guardian's signature--- Date-----------------------
Parent/guardian's name --- Time------------------------
(Please print your name)

I certify that I have followed all the study procedures in the S.O.P for obtaining consent.
Coordinator/researcher's signature--- Date----------
Coordinator/researcher's name -- Time----------
(Please print your name)

This is only necessary when the Guardian/Parent cannot read:

I have witnessed the information concerning this study being fully explained and understood by the Parent/Guardian and permission has been granted by the Parent/Guardian without being forced or coerced.
Witness' signature-----------------------------------Date----------------------
Witness' name--------------------------------------Time-----------------------
(Please print your name)

- A witness is a person not involved in the study or who is not involved in obtaining consent.

The Parent/Guardian's thumbprint as named above if he /she cannot write-----------

Parent or Guardian is to be given a copy of the signed consent form to keep.

KEMRI-Wellcome Research programme -Fomu ya Idhini.

Randomised, double blind, placebo-controlled trial of cotrimoxazole prophylaxis among HIV-uninfected children with severe malnutrition.

Mimi, nikiwa mlezi wa ....(jina la mtoto),nimepata maelezo ya utafiti huu. Nimeelewa yote yaliyosemwa na maswali yangu yalijibiwa yote nikaridhika. Ninaelewa kwamba ninaweza kubadili mawazo yangu wakati wowote na haitaathiri faida kwa mtoto wangu.

Tafadhali jaza visanduku vilivyo hapo chini inavyostahili: (*Tafadhali weka alama ya kukubali)*

☐ Ndiyo, **Nimekubali kuruhusu mtoto wangu kushiriki katika utafiti**.

☐ Ndiyo, **Nimekubali sampuli zihifadhiwe**.

☐ Ndiyo, **nimekubali sampuli zifanyiwe vipimo nchi ya ng'ambo**

Sahihi ya Mzazi/Mlezi ....Tarehe ....

Jina la mzazi/mlezi...Saa |__|__|: |__|__|
(tafadhali andika jina)

Ninathibitisha kwamba nimefuata mpangilio wote wa mradi katika SOP kwa kupata idhini.

Sahihi ya Mtafiti au mteule wake..Tarehe........................

Jina la Mtafiti au mteule wake...Saa |__|__|: |__|__|
(tafadhali andika jina)

<u>Hii ni muhimu tu kama mzazi/mlezi hawezi kusoma.</u>

*Nimeshuhudia kwamba habari zinazohusu utafiti huu zimeelezewa kikamilifu na kueleweka na mzazi/mlezi na kwamba idhini imetolewa na mzazi/mlezi bila kulazimishwa.

Sahihi ya shahidi ....**Tarehe**..................................

Jina la shahidi..**Saa**.......................................
(tafadhali andika jina)

(***Shahidi ni mtu ambaye hahusiki na mradi ama mfanyikazi ambaye hahusiki katika kupeana idhini.)**

Alama ya gumba ya mzazi kama ilivyotajwa hapo juu iwapo hawawezi kuandika

....

THUMB PRINT

MPE MZAZI/MLEZI NAKALA YAKE ILIYOWEKWA SAHIHI.

Fomu ya Idhini ya Mpango wa KEMRI-Wellcome

CTX Study (Kigiriama)

1. Mimi, here muimirizi wa ______________________________ (dzina ra muhoho) Nidzaelezwa utafiti uu; nidzaelewa gosini dzashomerwa na maswali ga kwangu gosi gajibiwa kuthosheka. Ninaelewa kala ninadima nikagaluza nia ya kwangu wakati wowosi na kaindadhuru mafanikio andigopata mwanangu.

☐ *Ika alama* **Ninakubali kuruhusu mwanangu ahusike kwenye utafiti uu.**
☐ *Ika alama* **Ninakubali sampuli ziikwe.**
☐ *Ika alama* **Ninakubali sampuli zisafirishwe.**

Sahihi ya Mzhazi / muimirizi **____________________________Tarehe: ________________**

Dzina ra Mzhazi / muimirizi **_____________________________ Saa: __________________**

(Tafadhali ndika dzinaro na herufi bomu)

Mimi nahakikisha mambo gosini ga haha dzulu gadzaelezwa kwa masumurira/ mlomo kwa mzhazi / muimirizi ni __________________________________ (dzina ra mtu wa KEMRI ahalaye idhini/ruhusa), na yuyu anaelewa hali na sababu ya mradhi na makubaliano ga muhoho kuhusishwa kwenye mradhi. Adzagerwa nafasi ya kuuza maswali ambago gajibiwa kuthosha.

Sahihi ya mchunguzi :__________________________Tarehe: _________________

Dzina: ___________________________ Saa: ____________________

(Tafadhali ndika dzinaro kwa herufi bomu)

Ni lazima thu kala mzhazi / msimamizi kadima kushoma.

2. Mimi Ninadhibitisha kwamba ujumbe urio kwenye fomu ya idhini idzaelezwa karakara, na ananyesa idzaelewekato ni mzhazi / muimirizi na kukala idhini baada ya kumbirizwa idzalazhwa huru ni mzhazi/ muimirizi.

Sahihi ya Shahidhi: _______________________________Tarehe: ____________________

Dzina ra shahidhi: ____________________________ Saa: _____________________

(*Tafadhali ndika dzinaro kwa herufi bomu)*

****Shahidhi ni akale ahuru na majaribio ama muhendi wa kazi wa KEMRI ariye kahusishirwe kwenye kupata idhini.***

Alama ya chala cha mzhazi adzehadzwa kala kamanya kundika: ______________________

MZHAZI / MUIMIRIRI KAMA RICHIRWA NAKALA IDZIYONGIZWA SAHIHI KUIKA.

Vitangulizi zha madzina ga muhusika: ___________

Nambari ya ombozero:_________ lugha: ______________________ siku/mwezi/mwaka.
